# Effects of a Fermented Dairy Drink Containing *Lacticaseibacillus paracasei* subsp. *paracasei* CNCM I-1518 (*Lactobacillus casei* CNCM I-1518) and the Standard Yogurt Cultures on the Incidence, Duration, and Severity of Common Infectious Diseases: A Systematic Review and Meta-Analysis of Randomized Controlled Trials

**DOI:** 10.3390/nu12113443

**Published:** 2020-11-10

**Authors:** Theresa Poon, Justine Juana, Daniel Noori, Stephanie Jeansen, Amira Pierucci-Lagha, Kathy Musa-Veloso

**Affiliations:** 1 Intertek Health Sciences, Inc., Suite 201, 2233 Argentia Road, Mississauga, ON L5N 2X7, Canada; justine.juana@intertek.com (J.J.); daniel.noori@intertek.com (D.N.); kathy.musa-veloso@intertek.com (K.M.-V.); 2 Danone Nutricia Research, RD 128, 91767 Palaiseau CEDEX, France; stephanie.jeansen@danone.com (S.J.); amira.pierucci@danone.com (A.P.-L.)

**Keywords:** *Lacticaseibacillus paracasei* subsp. *paracasei* CNCM I-1518, *Lactobacillus casei* CNCM I-1518, *Lactobacillus casei* DN-114 001, fermented dairy, fermented milk, probiotic, common infectious disease, respiratory infection, gastrointestinal infection, immune function

## Abstract

There is considerable interest in the role of probiotics in immune function. The objective of this systematic review and meta-analysis was to assess the effects of the consumption of a fermented dairy drink containing *Lacticaseibacillus paracasei* subsp. *paracasei* CNCM I-1518 (the previous taxonomic nomenclature was *Lactobacillus casei* CNCM I-1518, prior to the nomenclature change in April 2020) and the standard yogurt cultures (hereinafter referred to collectively as “FDD”) on common infectious diseases (CIDs) in generally healthy children and adults. Nine literature databases were searched, and nine randomized controlled trials from eight publications were eligible for inclusion. Combined effect sizes were determined for three metrics of CID incidence, two metrics of CID duration, and one metric of CID severity. Compared to the control, the consumption of the FDD resulted in (1) a significant reduction in the odds of experiencing ≥1 CID (odds ratio (OR) (with a 95% confidence interval (CI)): 0.81 (0.66, 0.98); *p* = 0.029); (2) a significant reduction in mean CIDs per subject (−0.09 (−0.15, −0.04); *p* = 0.001); and (3) a trend towards reduced risk in cumulative CIDs (relative risk (RR): 0.91 (0.82, 1.01); *p* = 0.082). The consumption of the FDD had no significant effect on CID duration or severity. Based on the studies conducted thus far, these results suggest that the FDD may reduce CID incidence in the general population.

## 1. Introduction

Common infectious diseases (CIDs) continue to contribute to the global non-fatal disease burden [1]. Though the term CID has not been formally defined by an authoritative or scientific body, CIDs are generally recognized to include respiratory tract infections (RTIs) and gastrointestinal tract infections (GITIs) [2]. According to the Global Burden of Diseases, Injuries, and Risk Factors Study, which includes global estimates of the incidence, prevalence, and years lived with disability for 354 conditions across 195 countries and territories from 1990 to 2017, the number of incident cases was 17.1 billion for upper respiratory tract infections (URTIs), 470,000 for lower respiratory tract infections (LRTIs), and 6.29 billion for infectious diarrheal diseases in 2017 [1]. Moreover, URTIs and infectious diarrheal diseases were consistently reported to be two of the three diseases with the highest global incidence rates between 1990 and 2017 [1]. Due to their high incidence rates, CIDs are associated with substantial direct medical costs, such as physician visits, hospitalizations, and medications, as well as indirect medical costs incurred from the loss of productivity and absenteeism from work or school, resulting in increased economic burden [3,4,5,6,7,8]. In addition, CIDs have been reported to have significant adverse effects on the quality of life of patients and their families [5,9,10,11,12].

The supplementation of the diet with probiotics has been shown to be a promising, preventive approach against various infections, which include RTIs and GITIs [13,14,15,16,17,18,19,20,21,22]. *Bifidobacteria* and *Lactobacilli* are the two most commonly used genera in probiotic studies [23]. Along with the standard yogurt cultures (i.e., *Lactobacillus bulgaricus* and *Streptococcus thermophilus*), *Lacticaseibacillus paracasei* subsp. *paracasei* CNCM I-1518, a well-characterized probiotic strain, is present in commercialized fermented dairy products that are marketed under brand names such as Actimel® and DanActive® [24]. In April 2020, the nomenclature of the genus *Lactobacillus* was revised, and *Lactobacillus paracasei* (or *Lactobacillus casei*) was renamed *Lacticaseibacillus paracasei* [25]; as such, prior to April 2020, *Lacticaseibacillus paracasei* subsp. *paracasei* CNCM I-1518 was referred to as *Lactobacillus casei* CNCM I-1518 or *Lactobacillus casei* DN-114 001. Though the mechanism of action has not yet been fully elucidated, the probiotic strain *L. paracasei* subsp. *paracasei* CNCM I-1518 has been demonstrated to prevent the adhesion and invasion of *Escherichia coli* in vitro [26], limit chemically-induced gut injuries [27,28], and enhance antimicrobial activity in vitro [29]. In addition, *L. paracasei* subsp. *paracasei* CNCM I-1518 has been reported to have a high survivability in the gastrointestinal tract of mice and humans, which is an important indication of the potential functionality of the probiotic strain [30,31,32]. Moreover, it has been demonstrated in in vitro and in vivo studies that *L. paracasei* subsp. *paracasei* CNCM I-1518 modulates the molecules involved in humoral and cell-mediated immune responses [33,34,35]. Furthermore, data from human studies have indicated that fermented dairy products containing *L. paracasei* subsp. *paracasei* CNCM I-1518 and the standard yogurt cultures may modulate biomarkers of immune function [36,37,38,39], as well as immune responses [24,39,40,41,42].

In recent years, several systematic reviews on the effects of probiotics on infectious diseases have been published; however, the probiotic strains investigated within each systematic review were variable [15,17,18,19,20,21,22]. Though these analyses attempted to isolate the effects of the individual strains whenever possible, limited information on *L. paracasei* subsp. *paracasei* CNCM I-1518 was available. Therefore, with increasing data available on *L. paracasei* subsp. *paracasei* CNCM I-1518, there is a need to fully understand the effects of foods containing the probiotic *L. paracasei* subsp. *paracasei* CNCM I-1518 on CIDs in the general population. Based on the literature available, it is hypothesized that *L. paracasei* subsp. *paracasei* CNCM I-1518 may reduce the risk of CIDs or ease the burden of CIDs in humans. Thus, the objective of this systematic review was to assess the effects of the consumption of a fermented dairy drink containing *L. paracasei* subsp. *paracasei* CNCM I-1518 and the standard yogurt cultures (hereinafter referred to collectively as FDD) on CIDs in generally healthy persons aged two years and older. In the current analysis, CIDs were defined to include URTIs (e.g., rhinosinusitis, pharyngitis, laryngitis, acute otitis media), LRTIs (e.g., acute bronchitis, bronchiolitis, pneumonia, tracheitis), and GITIs (e.g., diarrhea).

## 2. Materials and Methods

The research question was developed using the PICOS (Population, Intervention, Comparator, Outcomes, Study design) framework [43] (Table 1). The systematic review was conducted in accordance with the guidelines of the Preferred Reporting Items for Systematic Reviews and Meta-Analyses (PRISMA) statement [43].

### 2.1. Literature Search

Two literature searches were conducted in November and December 2019 using the electronic search tool ProQuest Dialog (ProQuest LLC). Nine literature databases were searched: Adis Clinical Trials Insight (Springer Healthcare), Allied and Complementary Medicine™ (The British Library), BIOSIS Previews® (Clarivate Analytics), CAB Abstracts (CAB International), Embase® (Elsevier B.V.), Foodline®: SCIENCE (Leatherhead Food Research), Food Science and Technology Abstracts® (IFIS), MEDLINE® (US National Library of Medicine), and National Technical Information Service (National Technical Information Service, US Department of Commerce).

Three sets of search terms were used to identify the exposure, health outcome, and study population. Exposure search terms comprised Actimel or fermented NEAR/3 (milk or yogurt or yogourt or yoghurt or drink or dairy) or “sour milk” or sour-milk or “*Lactobacillus casei*” or “*L. casei*” or “*L casei*” or “CNCM I-1518” or DN-114001 or “DN 114001” or “DN-114 001” or “DN 114 001” or ACTN06 or “*Lactobacillus paracasei*” or “*L. paracasei*” or “*L paracasei*” or “*Lactobacillus bulgaricus*” or “*L. bulgaricus*” or “*L bulgaricus*” or “*Lactobacillus delbrueckii* subsp. *bulgaricus*” or “*Lactobacillus delbrueckii* ssp. *bulgaricus*” or “*Lactobacillus delbrueckii* subspecies *bulgaricus*” or “*Streptococcus thermophilus*” or “*S. thermophilus*” or “*S thermophilus*” or “*Streptococcus salivarius* subsp. *thermophilus*” or “*Streptococcus salivarius* ssp. *thermophilus*” or “*Streptococcus salivarius* subspecies *thermophilus*." Of note, after the literature searches were conducted, the nomenclature of the genus *Lactobacillus* was revised in April 2020, and *Lactobacillus paracasei* (or *Lactobacillus casei*) was renamed as *Lacticaseibacillus paracasei* [25]. Health outcome search terms comprised "common infectious disease*” or CID or “respiratory tract infect*” or RTI or “respiratory infect*” or “gastrointestinal infect*” or GITI or “intestinal infection” or “enteric infection” or influenza or flu* or bronchitis or bronchiolitis or pneumonia or croup or gastroenteritis or norovirus or rotavirus or rhinovirus or diarrhoea or diarrhea. Study population search terms comprised men or women or man or woman or human or humans or subject or subjects or participant* or volunteer* or patient* or people or person* or individual* or student* or elder* or senior* or geriatric or older or adult* or teen* or adolescen* or child* or toddler* or boy or boys or girl or girls or pediatric or paediatric or clinical. The NEAR/3 command was used to search for two terms, in any order, separated by a maximum of three words (e.g., fermented NEAR/3 milk would have identified “fermented milk” and “milk product fermented with”). The asterisk was used to allow for flexibility on the word ending (e.g., adolescen* would have identified “adolescents” and “adolescence”). For an article to be identified, one search term from each of the three sets of keywords was required to appear, either in the title or abstract of the articles. No limitations with respect to the publication date or language were imposed.

### 2.2. Study Inclusion and Exclusion Criteria

The following inclusion criteria were applied: (1) the food studied was the FDD (which contained *L. paracasei* subsp. *paracasei* CNCM I-1518 and the standard yogurt cultures, *L. bulgaricus* and *S. thermophilus*); (2) the human intervention study was randomized and controlled; (3) the study population was comprised of generally healthy persons ≥2 years of age who did not have serious diseases (e.g., cardiovascular disease or cancer); (4) the incidence, duration, or severity of CIDs was assessed; (5) the independent effects of the investigational product could be isolated (e.g., the FDD was not co-administered with other bioactives known to affect the incidence, duration, and/or severity of CIDs); and (6) the full-length article was published in English in a peer-reviewed journal.

The following exclusion criteria were applied: (1) it was an animal or in vitro study; (2) the study population consisted predominantly of infants and children younger than 2 years of age (i.e., the proportion of subjects younger than 2 years of age was ≥80%); (3) the outcome was not a CID (e.g., the outcome was antibiotic- or radiation-induced diarrhea or allergic rhinitis); (4) the study was published in abstract form only or as a short communication (e.g., letter to the editor or commentary); (5) the publication was a secondary research study (e.g., systematic review or meta-analysis); and (6) the study was a kin publication to another study (i.e., the study results for the same population group were published in another journal). Though secondary research studies were excluded, the reference lists of systematic reviews or meta-analyses were screened to ensure the identification of all relevant studies. The filtration of the literature was conducted by one author (D.N.) and reviewed by another author (T.P.). Where there were discrepancies, additional authors (S.J. and K.M.-V.) provided input.

### 2.3. Data Extraction and Assessment of Study Quality

Data extracted from the studies included study design, study duration, country in which the study was conducted, sample size (initial and final), study population (e.g., gender, age, and health status), investigational products (e.g., composition and dosing instructions), CID-related endpoints (e.g., the definitions and methods used in their diagnosis and assessment), quantitative outcomes (e.g., incidence, duration, and severity), metrics of the outcomes assessed (e.g., cumulative days of CIDs versus mean days per CID episode), and statistical results between the active and control groups. 

The National Institutes of Health (NIH) tool for the quality assessment of controlled intervention studies was used for the assessment of study quality [44]. For three of the fourteen NIH quality criteria (i.e., criteria #6, #10, and #11), additional confounders pertinent to the assessment of CID-related outcomes were considered. For quality criterion #6 related to whether the groups were similar at baseline with respect to important characteristics that could affect outcomes, in addition to the general demographic characteristics (e.g., age and gender), the following characteristics at baseline were considered: (1) presence of CIDs; (2) influenza or rotavirus vaccination status; and (3) medication/supplement use (e.g., proton pump inhibitors). For quality criterion #10 related to whether other interventions were avoided or similar between groups (e.g., similar background treatments), the following other interventions during the study were considered: (1) the use of rescue medications/supplements (e.g., for colds, flu, or diarrhea); and (2) the consumption of other probiotics. For quality criterion #11 related to whether the outcomes were assessed using valid and reliable measures implemented consistently across all study participants, the following were considered: (A) the incidence of CIDs: (1) diagnosed by a physician/health professional; (2) if not diagnosed by a physician/health professional, type of symptoms listed (e.g., sneezing or runny nose); and (3) if not diagnosed by a physician/health professional, number and duration of symptoms used to define a CID episode (e.g., must have at least two symptoms within two consecutive days); (B) the duration of CIDs: how duration was determined (e.g., first to the last day of symptoms); and (C) the severity of CIDs: how severity was determined (e.g., scoring system: mild, moderate, or severe). Based on the overall assessment, the study was then rated as being of “poor,” “fair,” or “good” quality, as per the guidance provided by the NIH [44]. It should be noted that criteria #6, #10, and #11 were considered highly important in determining whether conclusions could be drawn from a study, and so a study could be rated as “poor quality” based on either of these three criteria even if all other quality appraisal criteria were adequately established. Study quality was independently appraised by two authors (T.P. and J.J.).

### 2.4. Statistical Analysis

Across all studies, data related to total CIDs were summarized; specifically, the outcomes (i.e., incidence, duration, and severity) and the metrics used to define these outcomes (e.g., for the duration of CIDs, cumulative days of CIDs versus mean days per CID episode) were tabulated. Comprehensive Meta-Analysis Software (Version 2.2.064, Biostat, Inc., Englewood, New Jersey, United States) was used to run all meta-analyses and generate forest plots for outcomes for which the same metric was reported in two or more studies. That is, combined estimates were determined for three metrics of CID incidence: (1) the relative risk (RR), (2) the difference in means in the number of CIDs per subject, and (3) the odds ratio (OR) for the number of subjects who experienced one or more CID (i.e., occurrence). Combined estimates were determined for two metrics of CID duration: (1) the difference in means in the cumulative days of CIDs amongst subjects with CIDs and (2) the difference in means in the days per CID episode amongst subjects with CIDs. For CID severity, one combined estimate was determined: the OR for the cumulative number of CIDs categorized as “severe” amongst subjects with CIDs. For each combined effect, 95% confidence intervals (CIs) were also generated. The studies that were combined varied with regard to several different factors (e.g., study population, duration, and/or country of conduct); thus, a random effects model was used, according to the methods described by DerSimonian and Laird [45]. The inverse of the variance was used as the weighting factor for all the meta-analyses in which the combined effect was a continuous variable (i.e., mean number of CIDs per subject, mean cumulative days of CIDs amongst subjects with CIDs, and mean days per CID episode amongst subjects with CIDs). Publication bias was assessed according to the trim and fill method developed by Duval and Tweedie [46]. With this method, missing studies are searched for and imputed, and then the combined effect is recomputed. Heterogeneity was assessed using the I^2^ statistic, which describes the percentage of variation across studies that cannot be attributed to chance [47,48]. I^2^ values of 25%, 50%, and 75% were considered to reflect low, moderate, and high heterogeneity, respectively [48]. The raw data for the meta-analyses were compiled by two authors (T.P. and J.J.), and the statistical analyses, including the meta-analyses, assessment of heterogeneity, assessment of publication bias, and generation of forest plots, were conducted by K.M.-V.

## 3. Results

### 3.1. Identification of Literature

**Figure 1:**
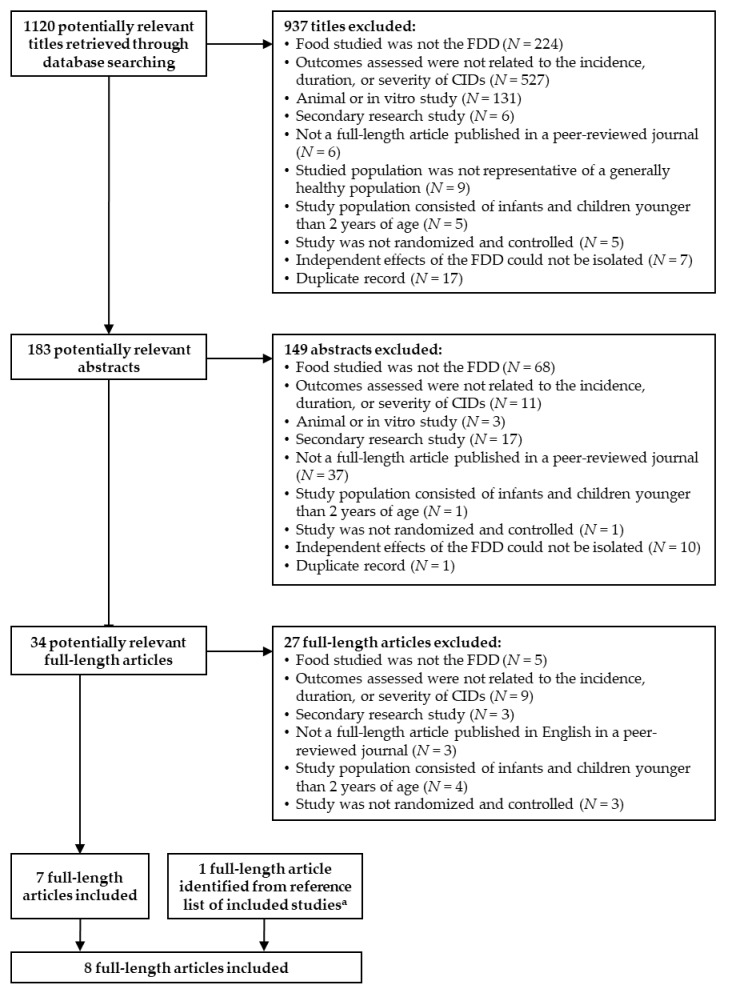
Flow chart of the literature search process. CID: common infection disease; FDD: fermented dairy drink containing *Lacticaseibacillus paracasei* subsp. *paracasei* CNCM I-1518 and the standard yogurt cultures. ^a^ Identified from the reference lists of the studies by Guillemard et al. [39,42].

The literature search resulted in the identification of 1120 titles, of which seven publications met all of the inclusion criteria and none of the exclusion criteria [2,24,39,40,41,42,49]. An additional study by Tiollier et al. [50] was identified in the reference lists of the studies by Guillemard et al. [39,42] and determined to meet all the inclusion criteria and none of the exclusion criteria. The study by Tiollier et al. [50] was not identified in the literature search likely because neither the title nor abstract contained any of the keywords for subject population. The publication by Boge et al. ([40], pilot and confirmatory studies) consisted of a pilot and a confirmatory study. Thus, the effects of the FDD on CIDs were assessed in a total of nine studies from eight publications (Figure 1). Of the nine studies, two were conducted in generally healthy children [2,24], three were conducted in adults [39,49,50], and four were conducted in the elderly ([40], pilot and confirmatory studies) and [41,42] (Table 2).

### 3.2. Children

Across two studies, the effects of the FDD on CIDs were assessed in generally healthy boys and girls aged 3–6 years residing in the United States [24] or Russia [2] (Table 2). In each study, the final sample size was approximately 600 subjects. Both studies were randomized, double-blinded, and placebo-controlled. In both studies, the dose of the FDD was 200 g or mL/day, which provided at least 2 × 10^10^ colony forming units (CFU)/day of *L. paracasei* subsp. *paracasei* CNCM I-1518. The FDD was consumed in two divided doses (2 × 100 g), daily, for 12 weeks [2] or once per day for 13 weeks [24].

According to the NIH quality appraisal tool, study quality was rated as good for the study by Prodeus et al. [2] and poor for the study by Merenstein et al. [24] (Table 2). The rationale for the ratings of the individual quality criteria is presented in Supplementary Table S1. With respect to the confounders considered pertinent to this assessment on CID-related outcomes, all were addressed in the study by Prodeus et al. [2] (Table 3). In contrast, several confounders were not accounted for in the study by Merenstein et al. [24]—the children’s vaccination status for influenza or rotavirus at baseline was not reported, and the CIDs were self-reported by the children’s parents and not diagnosed by a physician/health professional. In addition, in the study by Merenstein et al. [24], there was a statistically significant difference between groups in the number of study products consumed (i.e., 6.5 and 6.1 drinks/week in the active and control groups, respectively; *p* = 0.004).

### 3.3. Adults

Across three studies, the effects of the FDD on CIDs were assessed in generally healthy adults residing in Germany [39], Israel [49], or France [50] (Table 2). Both men and women were included in one study [39], whereas young men undergoing military training were included in the other two studies [49,50]. The final sample size was approximately 50 [50], 500 [49], or 1000 subjects [39]. While all three studies were randomized and placebo-controlled, two were double-blinded [39,50], and one was single-blinded [49]. In the study by Pereg et al. [49], the dose of the FDD was 100 mL/day, which provided 1 × 10^10^ CFU/day of *L. paracasei* subsp. *paracasei* CNCM I-1518. In the study by Guillemard et al. [39], the dose of the FDD was 200 g/day, which provided at least 2 × 10^10^ CFU/day of *L. paracasei* subsp. *paracasei* CNCM I-1518. In the third study, the dose of the FDD was reported to be 300 mL/day; however, the corresponding dose of *L. paracasei* subsp. *paracasei* CNCM I-1518 provided by the FDD was not reported [50]. The FDD was consumed three times per day (3 × 100 mL) for 4 weeks [50], once per day for 8 weeks [49], or twice per day (2 × 100 g) for 12 weeks [39].

Study quality was rated as good in one study [39] and poor in two studies [49,50] (Table 2; Supplementary Table S1). With respect to the confounders considered pertinent to this assessment on CID-related outcomes, the majority were addressed in the study by Guillemard et al. [39] (Table 3). In contrast, in both the studies conducted in young men undergoing military training [49,50], the majority of confounders were not considered, including all three confounders at baseline, the use of rescue medications during the study, and the self-reporting of CIDs without diagnosis by a physician/health professional.

### 3.4. Elderly

Across four studies, the effects of the FDD on CIDs were assessed in free-living [41,42] or institutionalized ([40], pilot and confirmatory studies) elderly men and women aged >60 years residing in Europe (Table 2). The final sample size was approximately 75 ([40], pilot and confirmatory studies), 200 ([40], pilot and confirmatory studies), 350 [41], or 1000 [42]. Three of the studies were randomized, double-blinded, and placebo-controlled ([40], pilot and confirmatory studies) and [42], whereas the study by Turchet et al. [41] was randomized and open-label, such that subjects in the control group did not receive any study product. In all studies, the dose of the FDD was 200 g or mL/day. The corresponding dose of *L. paracasei* subsp. *paracasei* CNCM I-1518 provided by the FDD was 2 × 10^10^ CFU/day [41,42] or not reported ([40], pilot and confirmatory studies). The FDD was consumed twice per day (2 × 100 g or mL) for either 3 [41], 7 ([40], pilot and confirmatory studies), 12 [42], or 13 weeks ([40], pilot and confirmatory studies).

Study quality was rated as good in one study [42], fair in one study ([40], pilot and confirmatory studies), and poor in two studies [40,41] (Table 2; Supplementary Table S1). With respect to the confounders considered pertinent to this assessment on CID-related outcomes, all were addressed in the study by Guillemard et al. [42] (Table 3). In contrast, several important confounders were not accounted for in the other three studies. In the study by Turchet et al. [41], there was a statistically significant difference between groups in the mean age of the elderly subjects at baseline (i.e., 67.1 ± 6.0 and 69.3 ± 5.6 years in the active and control groups, respectively; *p*-value was not reported). Moreover, 25% (45 of 180) of the subjects in the FDD group experienced dyspepsia during the study, and so the dosing regimen was reduced from two bottles to one bottle/day of the FDD for these subjects [41]. Furthermore, the use of rescue medications during the study was not reported, and subjects were allowed to consume up to two additional servings of other fermented dairy products per week [41]. In the pilot and confirmatory studies by Boge et al. ([40], pilot and confirmatory studies), the use of rescue medications during the study was not reported on. Notably, as the CID outcomes were reported as adverse events and not as primary or secondary outcomes, details related to the diagnosis of and methods used to assess the incidence, duration, and severity of CIDs were not reported ([40], pilot and confirmatory studies). Insufficient data were provided in the confirmatory study ([40], pilot and confirmatory studies) to permit an evaluation of the differential drop-out rate between the groups and whether it was ≤15%. Thus, although the majority of quality criteria were accounted for in the confirmatory study by Boge et al. ([40], pilot and confirmatory studies), the aforementioned study limitations were considered as fatal flaws—hence, the quality rating of poor.

### 3.5. Meta-Analyses

The metrics used to define efficacy, even across a single outcome, varied widely across the studies (Table 4). Due to the variability of the data, there was a scarcity of data appropriate for combining within each of the age groups (i.e., children, adults, and elderly). Thus, data were combined across the age groups in order to permit the conduct of meta-analyses. None of the meta-analyses presented in this systematic review included data from four of the six studies for which the quality was rated as poor [40,49,50] or fair ([40], pilot and confirmatory studies). This is because, as per Table 4, numerical results were not reported in the publications to permit the inclusion of these studies ([40], pilot and confirmatory studies) and [50] or the outcomes assessed in the study were unique and not reported on in the other studies, thereby precluding the ability to combine results across studies [49]. The only two studies included in the meta-analyses for which the quality was rated as poor were the studies by Merenstein et al. [24] and Turchet et al [41].

**Figure 2:**
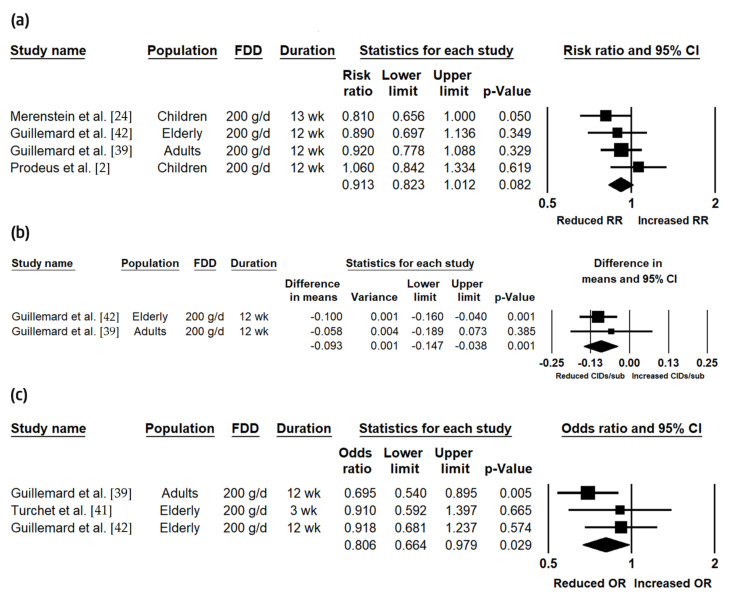
Effects of the fermented dairy drink containing *Lacticaseibacillus paracasei* subsp. *paracasei* CNCM I-1518 and the standard yogurt cultures (FDD) compared to the control on: (**a**) incidence relative risk (RR); (**b**) the mean number of common infectious diseases (CIDs) per subject; (**c**) the odds of experiencing ≥1 CID. CI: confidence interval; g/d: g/day; OR: odds ratio; wk: weeks.

The effects of the FDD on the incidence of CIDs were assessed across three meta-analyses (Table 5; Figure 2). First, the incidence of CIDs was presented as the RR based on the number of cumulative CIDs in four studies, which were conducted across all age groups [2,24,39,42]. After combining the results from these four studies, the consumption of the FDD was associated with a trend toward a reduced risk in the number of cumulative CIDs compared to placebo (RR (95% CI) = 0.91 (0.82, 1.01); *p* = 0.082) (Figure 2A). Second, the incidence of CIDs, defined as the mean number of CIDs per subject, was combined across two studies, one of which was conducted in adults and the other in elderly [39,42]. Accordingly, the consumption of the FDD significantly reduced the mean number of CIDs per subject compared to placebo (−0.09 (−0.15, −0.04); *p* = 0.001) (Figure 2B). Third, the incidence of CIDs, defined as the number of subjects who experienced ≥1 CID, was combined across three studies, which were conducted in adults and the elderly [39,41,42]; the consumption of the FDD significantly reduced the odds of experiencing ≥1 CID compared to a control (OR = 0.81 (0.66, 0.98); *p* = 0.029) (Figure 2C). Though there were insufficient data to assess publication bias in the second meta-analysis, there was no indication of publication bias in the other meta-analyses, and heterogeneity was low and not statistically significant in all the analyses.

**Figure 3:**
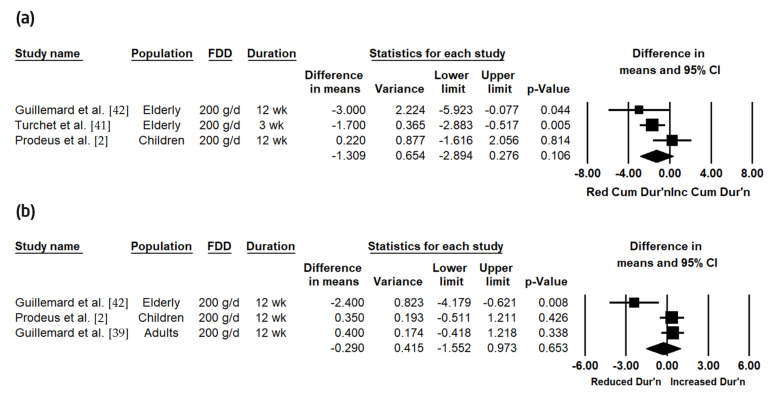
Effects of the fermented dairy drink containing *Lacticaseibacillus paracasei* subsp. *paracasei* CNCM I-1518 and the standard yogurt cultures (FDD) compared to the control on: (**a**) the mean cumulative days of common infectious diseases (CIDs) amongst subjects with CIDs; (**b**) the mean number of days per CID episode amongst subjects with CIDs. CI: confidence interval; cum dur’n: cumulative duration; dur’n: duration; g/d: g/day; wk: weeks.

The effects of the FDD on the duration of CIDs were assessed across two meta-analyses (Table 5; Figure 3). First, the duration of CIDs, defined as the mean cumulative days of CIDs amongst subjects with CIDs, was combined across three studies, which were conducted in children and the elderly [2,41,42]; the mean cumulative days of CIDs amongst subjects with CIDs was not significantly different between the active and control (−1.31 (−2.89, 0.28) days; *P* = 0.106) (Figure 3A). Heterogeneity was moderate and trended toward significance (*I^2^* = 55.85, *p* = 0.079). Using the trim-and-fill method of Duval and Tweedie [46] for the assessment of publication bias, one study was found to be missing to the right of the combined effect; with this study imputed, the recomputed combined effect was -0.86 (−2.45, 0.72) days. Second, the duration of CIDs, defined as the mean number of days per CID episode amongst subjects with CIDs, was combined across three studies, which were conducted across all age groups [2,39,42]; accordingly, the mean number of days per CID episode amongst subjects with CIDs was not significantly different between the active and placebo groups (−0.29 (−1.55, 0.97) days; *p* = 0.653) (Figure 3B). Heterogeneity was high and statistically significant (*I^2^* = 76.28, *p* = 0.015). There was no indication of publication bias.

**Figure 4:**
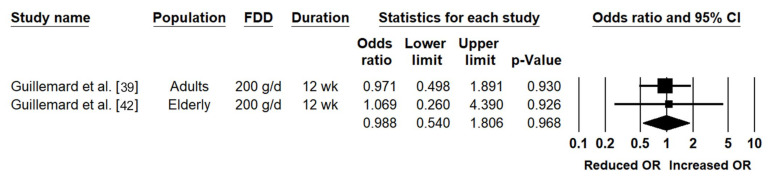
Effects of the fermented dairy drink containing *Lacticaseibacillus paracasei* subsp. *paracasei* CNCM I-1518 and the standard yogurt cultures (FDD) compared to the control on the odds of experiencing common infection diseases (CIDs) categorized as “severe” amongst subjects with CIDs. CI: confidence interval; g/d: g/day; OR: odds ratio; wk: weeks.

The severity of CIDs was presented as the cumulative number of CIDs categorized as “severe” amongst subjects with CIDs in two studies, which were conducted in adults and the elderly [39,42]. After combining the results from these two studies, the cumulative number of CIDs categorized as “severe” amongst subjects with CIDs was not significantly different between the active and placebo (OR = 0.99 (0.54, 1.81); *p* = 0.968) (Table 5; Figure 4). Heterogeneity was low and not statistically significant (*I^2^* = 0.00, *p* = 0.903), and publication bias could not be assessed due to insufficient data.

## 4. Discussion

Based on the results of the meta-analyses presented herein, the consumption of the FDD may reduce the incidence of CIDs; this was determined via three metrics (i.e., RR, mean number of CIDs per subject, and OR for the number of subjects who experienced ≥1 CID). Compared to a placebo or control, the consumption of the FDD was associated with a significant reduction in the odds of experiencing ≥1 CID (OR = 0.81 (0.66, 0.98); *p* = 0.029). Though a significant reduction in the mean number of CIDs per subject (−0.09 (−0.15, −0.04); *p* = 0.001) was observed following the consumption of the FDD compared to placebo, the magnitude of this reduction was small and difficult to interpret. In addition, the consumption of the FDD compared to placebo was associated with a trend towards a reduced risk in the number of cumulative CIDs (RR = 0.91 (0.82, 1.01); *p* = 0.082).

In contrast, the results of the meta-analyses did not suggest a beneficial effect for the FDD compared to a control in reducing the duration of CIDs; this was determined via two metrics (i.e., amongst subjects with CIDs, mean cumulative days of CIDs and mean days per CID episode). Similarly, a beneficial effect in reducing the OR for the number of CIDs categorized as “severe” amongst subjects with CIDs was not observed with the consumption of the FDD compared to placebo. Of note, the latter outcome was not indicative of a lack of efficacy in improving the severity of CIDs; rather, the metric that was assessed pertained specifically to CIDs that were categorized as “severe.” Indeed, in both studies in which this metric was assessed [39,42], severity was assessed using a three-point scale (i.e., mild, moderate, and severe), and results pertaining to CIDs categorized as “mild” or “moderate” were not reported.

Though several systematic reviews and meta-analyses on the effects of probiotics on infectious diseases have been published recently, the outcomes included in these meta-analyses differ from those assessed in our systematic review [18,20,51,52]. In general, it appears that the consumption of *L. paracasei* subsp. *paracasei* CNCM I-1518 is associated with beneficial effects across a broad spectrum of health outcomes, including the prevention of *Clostridium difficile* infections [18,51], the eradication of *Helicobacter pylori* infection amongst children in conjunction with triple therapy (i.e., proton pump inhibitor and two antibiotics) [20], and the prevention of antibiotic-associated diarrhea amongst adults [52]. Moreover, other published systematic reviews and meta-analyses have included studies in which multiple probiotic strains were investigated [17,22]. For example, given that the genus *Lactobacillus* alone is taxonomically complex wherein different species exhibit varying anti-infectious properties [53,54], the collective reporting of diverse *Lactobacillus* species obscures these differences, and the resulting generalizations may not be appropriate [52]. As such, the comparability of the results presented in our systematic review against those reported in the published literature is limited. Notably, this is the first systematic review and meta-analysis in which the effects of an FDD containing *L. paracasei* subsp. *paracasei* CNCM I-1518 and the standard yogurt cultures on CIDs were assessed. 

While the microbiota of the gastrointestinal tract is the most extensively studied microbial ecosystem, there is increasing interest in the microbiotas of other sites, such as the lungs, and their role in host homeostasis and disease development [55,56,57]. In contrast to the gut microbiota, the lung microbiota represents a considerably lower biomass and are hypothesized to consist of transient microbiota recolonized through aspiration, as opposed to resident and viable microorganisms [55,57]. Though the gut and lungs are anatomically distinct and exist in different environments, there is growing evidence for the interaction or cross-talk between these respective microbiotas, termed the “gut–lung axis” [55,57]. It appears that when the gut microbiota is disturbed, such as when infection occurs, the normal microbiota-derived signals are altered, which leads to a modified immune response [55]. For example, in mice with pathogenic bacterial infection of the lungs, exposure to gut microbiota-derived ligands (e.g., lipopolysaccharide or peptidoglycan) resulted in improved immune responses [58,59]. In addition, metabolites produced by gut bacteria, such as short-chain fatty acids, can enter the systemic circulation and modulate the immune response in the lungs [55,56,60]. While the exact mechanisms by which the gut microbiota modulate lung immune responses are still under investigation [55,56,57], it is clear that the bacterial components and metabolites in the gut and lungs have the capacity to modulate local and systemic immunity, as well as that specific taxa are able to influence the pathogenesis of respiratory infections and diseases (e.g., asthma) [55,56,57,60,61,62,63].

Though the precise mechanism of *L. paracasei* subsp. *paracasei* CNCM I-1518 on systemic immunity has not yet been fully elucidated, it has been postulated that *L. paracasei* subsp. *paracasei* CNCM I-1518 potentially influences systemic immunity via three modes of action: the modulation of the gut microbiota, epithelial barrier, and local mucosal immune response. First, within the context of colonization resistance whereby the gut microbiota protects itself from foreign microbes through microbe–microbe or microbe–host interactions [64,65], *L. paracasei* subsp. *paracasei* CNCM I-1518 is thought to contribute to gut microbial balance in mice [66], as well as infants and young children [67]. Second, *L. paracasei* subsp. *paracasei* CNCM I-1518 is thought to strengthen epithelial barrier function [26,27,28,29,68] by inhibiting pathogenic strain adhesion and growth, as suggested by in vitro studies [26,69,70,71]. For example, *L. paracasei* subsp. *paracasei* CNCM I-1518 inhibited the adhesion of enteropathogenic *E. coli* (EPEC) onto cultured epithelial cells [26], and it also inhibited the increase in paracellular permeability of cells infected with EPEC [70]. In vitro and in vivo data in rodents have shown that *L. paracasei* subsp. *paracasei* CNCM I-1518 stimulates the maturation and differentiation of intestinal epithelial cells [29,72,73]. Mice administered a fermented milk containing *L. paracasei* subsp. *paracasei* CNCM I-1518 and the standard yogurt cultures were observed to have a significantly increased production of goblet cells responsible for maintaining the integrity of the protective mucus layer [74].

Third, *L. paracasei* subsp. *paracasei* CNCM I-1518 is thought to modulate the mucosal immune response [33,75,76]. For example, it has been shown in in vitro studies that, in the presence of molecules that mimic potential pathogens or danger signals, *L. paracasei* subsp. *paracasei* CNCM I-1518 was able to interact with dendritic cells and increase their ability to promote T helper type 1 responses [75,76]. In several studies conducted in mice, the administration of a fermented milk containing *L. paracasei* subsp. *paracasei* CNCM I-1518 and the standard yogurt cultures resulted in increased levels of immunoglobulin A+ and CD8+ and CD4+ T cells in the small intestine [74,77], as well as the increased production of interleukin-6 by intestinal epithelial cells, which is a cytokine that plays an important role in initiating and maintaining the interaction between the intestinal epithelial cells and intestinal immune cells [74,78,79].

As *L. paracasei* subsp. *paracasei* CNCM I-1518 has been shown to survive in the human gastrointestinal tract [31,32,68] where it is thought to exert its beneficial effect, it is hypothesized that *L. paracasei* subsp. *paracasei* CNCM I-1518 delivers probiotic effectors to immune cells located in the intestine. As such, in addition to the discussed in vitro and in vivo studies, the effects of the consumption of a fermented dairy product containing *L. paracasei* subsp. *paracasei* CNCM I-1518 and the standard yogurt cultures on systemic immune responses were investigated in several randomized controlled trials, which may explain the beneficial effects observed with respect to CIDs. For example, amongst subjects who experienced at least one CID, blood leukocyte, neutrophil, and natural killer (NK) cell counts were significantly increased from baseline in the group consuming the FDD compared to the placebo group [39]. Given that these immune cells are known to be involved in antibacterial and antiviral responses, this immune-modulation effect triggered by the FDD may explain the observed beneficial effect on CIDs in the active group. Of interest, other studies have demonstrated that the consumption of the FDD helps to modulate immunological biomarkers (e.g., NK cells) in students under academic examination stress [37], athletes subjected to an exercise stress test [36], and lactating women who had recently delivered [38]. In the study by Boge et al. ([40], pilot and confirmatory studies), the consumption of the FDD increased specific antibody responses to influenza vaccination in the elderly. It is also possible that other potentially bioactive ingredients (e.g., peptides, fatty acids, and enzymes) produced during the fermentation of the diary product may contribute to the observed benefits [79].

The main limitation associated with our meta-analysis is the combining of data across different age groups, which was necessitated by the variability in the metrics reported across studies and the resulting scarcity of data appropriate for combining within each of the age groups. It is acknowledged that certain age groups, including young children under five years of age and the elderly, are at an increased risk of certain types of CIDs, such as influenza [80]. Nevertheless, the World Health Organization recognizes that all age groups remain susceptible to CIDs [80]. Thus, while the approach would not have been ideal if there was an abundance of data, the combining of data across the age groups in the meta-analyses remains valid given the limitations of the current evidence base. Notwithstanding, the results presented herein should be interpreted with an understanding that the results were obtained from a limited number of studies. In addition, the comparisons in all of the studies included in the meta-analyses were between the FDD containing *L. paracasei* subsp. *paracasei* CNCM I-1518 and the standard yogurt cultures versus a non-fermented acidified dairy drink (i.e., void of the standard yogurt cultures), with the exception of the study by Turchet et al. [41] in which the comparison was to no product. Thus, the results of the meta-analyses, particularly on CID incidence, should be interpreted within the context of the comparison to a non-fermented acidified dairy drink.

With this being the first systematic review and meta-analysis to investigate the effects of an FDD containing *L. paracasei* subsp. *paracasei* CNCM I-1518 and standard yogurt cultures, our aim was to focus the assessment on the critical outcomes that are of clinical significance—that is on the incidence, duration, and severity of total CIDs. Outcomes related to CID subcategories, including URTIs, LRTIs, and GITIs, were assessed in the majority of studies included in the assessment; however, the metrics for URTIs, LRTIs, and GITIs were incongruent across the studies and did not allow for the combining of results (e.g., cumulative days of GITIs amongst subjects with GITIs [42] versus cumulative days of GITIs amongst all subjects [39]). The adequate reporting and presentation of the results for these subcategories of CIDs will facilitate data combining in future systematic reviews and meta-analyses. In addition, it would be interesting for future systematic reviews and meta-analyses to examine other outcomes that may be helpful in supporting the beneficial clinical effects of the FDD, including, for example, outcomes related to fever, medication use, quality of life, and absenteeism due to sickness [2,24,39,41,42,49]. 

In conducting this systematic review and meta-analysis, it became apparent that there are challenges in conducting, interpreting, and appraising the quality of studies specifically on clinical outcomes of immune function. To ensure that the main sources of bias (e.g., selection, performance, detection, attrition, and reporting) were appraised, we opted to use the NIH tool for the quality assessment of controlled intervention studies [44]. To ensure the absence of confounding, a list of potential confounders relevant to clinical studies of immune function that we considered to be of importance was applied through three quality criteria (i.e., #6, #10, and #11) of the NIH tool (Table 3). These immune-specific confounders are important, such that their inadequate consideration within a study may decrease confidence in the results of a study. For example, the consideration of between-group comparability with respect to the subjects’ vaccination status, in this case for influenza or rotavirus, at baseline may increase confidence in the study results because vaccinations may influence the incidence of CIDs. In addition, the consideration of between-group comparability with respect to medication and supplement use at baseline may increase confidence in the study results because certain compounds may exert different effects on immune function; for example, the use of proton pump inhibitors has been associated with increased risk of various adverse effects in older adults, including *C. difficile* infection and community-acquired pneumonia [81]. Similarly, the consideration of between-group comparability with respect to rescue medication use (e.g., for colds or diarrhea) during the study may increase confidence in the study results because these medications may alter the duration of a CID and/or the subject’s perceived severity of a CID. We acknowledge that publications themselves are subject to bias given that all data may not be reported. Interestingly, the application of these criteria in the quality appraisal tool helped to differentiate studies in which these variables were addressed from those in which they were not. Furthermore, the methodological validity of the methods used to assess the outcomes related to the incidence, duration, and severity of CIDs is essential in understanding whether the study was susceptible to potential confounding. For example, although studies of this nature conducted in children have utilized parent-reported outcomes, the diagnosis of a CID by a health professional should be considered in order to minimize error and ensure consistency in reporting across subjects. While we recognize that no tool exists to specifically appraise the quality of clinical immune function studies, nor do any exist for other types of health outcomes, perhaps such a tool or, at the very least, a checklist of additional study considerations is needed for complex studies such as clinical immune function studies.

## 5. Conclusions

Overall, the results of this systematic review and meta-analysis contribute to the understanding of the beneficial effects of foods containing the probiotic *L. paracasei* subsp. *paracasei* CNCM I-1518 on CIDs in the general population; specifically, there is evidence, albeit from a limited number of studies, that FDDs containing *L. paracasei* subsp. *paracasei* CNCM I-1518 and standard yogurt cultures may reduce the incidence of CIDs. Moreover, this systematic review and meta-analysis highlights the challenges in conducting, interpreting, and appraising the quality of studies specifically on the clinical outcomes of immune function, as well as potential confounders specific to these types of studies. Based on the findings reported in this systematic review and meta-analysis, there appears to be a need for better guidance with regard to the proper design of clinical immune function studies, the standardization of clinical outcomes in these studies, and criteria that must be considered in the quality appraisal of these studies. With better guidance, it is hoped that the quality of future clinical immune function studies may be improved, such that they can be analyzed collectively in future systematic reviews and meta-analyses. 

## Figures and Tables

**Table 1:** Population, Intervention, Comparator, Outcomes, Study design (PICOS) framework.

Element	Description
Population	Generally healthy children and adults aged 2 years and older
Intervention	FDD, defined as a fermented dairy drink containing *Lacticaseibacillus paracasei* subsp. *paracasei* CNCM I-1518 and the standard yogurt cultures (i.e., *Lactobacillus bulgaricus* and *Streptococcus thermophilus*)
Comparator	No placebo Placebo (e.g., non-fermented dairy product)
Outcome	Incidence, duration, or severity of common infectious diseases, including upper respiratory tract infections (e.g., rhinosinusitis, pharyngitis, laryngitis, acute otitis media), lower respiratory tract infections (e.g., acute bronchitis, bronchiolitis, pneumonia, tracheitis), and gastrointestinal tract infections (e.g., diarrhea)
Study design	Randomized, controlled trial

**Table 2:** Key characteristics of included studies (*n* = 9 studies from 8 publications).

Reference	Study Design	Study Population		Interventions			Study Quality ^a^
		Sample Size Country of Conduct	Health Status at Baseline	Active	Control	Pattern of Consumption	
**Children (*n* = 2 Studies)**							
Merenstein et al. [24]	R, DB, PC, P Run-in: NR Intervention: 13 wk Follow-up: NR	*n*_i_ = 638 (329 M, 309 F) *n*_f_ = 636 (gender distribution NR) ITT = 638 PP = 564 ^b^ Mean age = 4.9 y U.S.	Healthy children, aged 3–6 y; vaccination status NR	200 mL/day FDD (1 × 10^8^ CFU/g *Lacticaseibacillus paracasei* subsp. *paracasei* CNCM I-1518; >1 × 10^7^ CFU/g yogurt cultures^c^)	200 mL/day non-fermented, acidified diary drink (assumed without standard yogurt cultures)	200 mL once per day (time of day NR)	Poor
Prodeus et al. [2]	R, DB, PC, P Run-in: NR Intervention: 12 wk Follow-up: 4 wk	*n*_i_ = 600 (325 M, 274 F, 1 NR) *n*_f_ = 584 (gender distribution NR) ITT = 599 ^d^ PP = 578 ^e^ Mean age = 4 y Russia	Healthy children aged 3–6 y; 32% of subjects were vaccinated against influenza during the previous year	200 g/day FDD (≥1 × 10^8^ CFU/g *L. paracasei* subsp. *paracasei* CNCM I-1518; ≥1 × 10^7^ CFU/g yogurt cultures^c^)	200 g/day non-fermented, acidified dairy drink without *Lactobacilli* and *Streptococcus thermophilus*	100 g twice per day (morning, afternoon)	Good
**Adults (*n* = 3 Studies)**							
Guillemard et al. [39]	R, DB, PC, P Run-in: 2 wk Intervention: 12 wk Follow-up: 4 wk	*n*_i_ = 1000 (435 M, 565 F) *n*_f_ = 962 (gender distribution NR) ITT = 1000 PP = 900 ^f^ Mean age = 32.2 y Germany	Healthy adults; 6.2% of subjects were vaccinated against influenza at study inclusion	200 g/day FDD (≥1 × 10^8^ CFU/g *L. paracasei* subsp. *paracasei* CNCM I-1518; ≥1 × 10^7^ CFU/g yogurt cultures^c^)	200 g/day non-fermented, acidified dairy drink (assumed without standard yogurt cultures)	100 g twice per day (breakfast, dinner)	Good
Pereg et al. [49]	R, SB, PC, P Run-in: NR Intervention: 8 wk (6 days/wk) Follow-up: NR	*n*_i_ = 541 M *n*_f_ = 502 M ITT = NA PP = 502 Mean age = 18.5 y Israel	Healthy adults residing in military camp; vaccination status NR	100 mL/day FDD (1 × 10^8^ CFU/mL *L. paracasei* subsp. *paracasei* CNCM I-1518; yogurt cultures and dose NR)	100 mL/day non-probiotic yogurt without live bacteria	100 mL once per day (time of day NR)	Poor
Tiollier et al. [50]	R, DB, PC, P Run-in: 3 wk Intervention: 4 wk^g^ Follow-up: 1 wk	*n*_i_ = *n*_f_ = 47 M ^h^ ITT = PP = 47 ^h^ Mean age = 21 ± 0.4 y France	Adults in good mental and physical condition undergoing army training; vaccination status NR	300 mL/day FDD (*L. paracasei* subsp. *paracasei* CNCM I-1518 dose NR; yogurt cultures and dose NR)	300 mL/day non-fermented milk (assumed without standard yogurt cultures)	100 mL three times per day (time of day NR)	Poor
**Elderly (*n* = 4 Studies)**							
Boge et al. ([40], pilot and confirmatory studies) pilot study	R, DB, PC, P Run-in: 1 to 4 wk Intervention: 7 wk Follow-up: 18.5 wk	*n*_i_ = 86 (30 M, 56 F) *n*_f_ = 75 (gender distribution NR) ITT = 86 PP = NA Mean age = 83.6 y France	Healthy elderly aged ≥70 y residing in nursing homes; all subjects were vaccinated against influenza 4 wk after product consumption	200 g/day FDD (*L. paracasei* subsp. *paracasei* CNCM I-1518 dose NR; yogurt culture dose NR ^c^)	200 g/day non-fermented acidified dairy drink (milk)	100 g twice per day (time of day NR)	Fair
Boge et al. ([40], pilot and confirmatory studies) confirmatory study	R, DB, PC, P Run-in: 1 to 4 wk Intervention: 13 wk Follow-up: 12.5 wk	*n*_i_ = 241 (74 M, 148 F, 19 NR) *n*_f_ = 195 (gender distribution NR) ITT = 222 ^i^ PP = NA Mean age = 84.6 y France	Healthy elderly aged ≥70 y residing in nursing homes; all subjects were vaccinated against influenza 4 wk after product consumption	200 g/day FDD (*L. paracasei* subsp. *paracasei* CNCM I-1518 dose NR; yogurt culture dose NR ^c^)	200 g/day non-fermented acidified dairy drink (milk)	100 g twice per day (time of day NR)	Poor
Guillemard et al. [42]	R, DB, PC, P Run-in: 2 wk Intervention: 12 wk Follow-up: 4 wk	*n*_i_ = 1072 (400 M, 672 F) *n*_f_ = 1026 (gender distribution NR) ITT = 1072 PP = 864 ^j^ Median age = 76.0 y France	Healthy, free-living elderly aged ≥70 y; all subjects were vaccinated against influenza ≥14 days before study inclusion	200 g/day FDD (≥1 × 10^8^ CFU/g *L. paracasei* subsp. *paracasei* CNCM I-1518; ≥1 × 10^7^ CFU/g yogurt cultures ^c^)	200 g/day non-fermented, acidified dairy drink (assumed without standard yogurt cultures)	100 g twice per day (breakfast, dinner)	Good
Turchet et al. [41]	R, OL, C, P Run-in: NR Intervention: 3 wk Follow-up: NR	*n*_i_ = 360 (119 M, 241 F) *n*_f_ = 358 (gender distribution NR) ITT = 360 PP = NA Mean age = 68.2 y Italy	Healthy, free-living elderly aged >60 years; 82% of subjects were vaccinated against influenza 3 months before study	200 mL/day FDD (1 × 10^8^ CFU/mL *L. paracasei* subsp. *paracasei* CNCM I-1518; yogurt cultures and dose NR)	No product	100 mL twice per day (time of day NR)	Poor

C: controlled; CFU: colony forming units; DB: double-blind; F: female; FDD: fermented dairy drink containing *L. paracasei* subsp. *paracasei* CNCM I-1518 and the standard yogurt cultures; ITT: intention-to-treat; M: male; NA: not applicable; nf: final sample size of study completers; ni: initial sample size of subjects randomized; NR: not reported; OL: open-label; P: parallel; PC: placebo-controlled; PP: per protocol; R: randomized; SB: single-blind; U.S.: United States; wk: weeks; y: years. ^a^ According to the National Institutes of Health tool for the quality assessment of controlled intervention studies, study quality could be rated as good, fair, or poor [44]. ^b^ A total of 74 subjects were not included in the PP analysis due to at least one major protocol deviation (22 in probiotic group and 52 in control group). ^c^ Two cultures commonly used in yogurt, *S. thermophilus* and *Lactobacillus delbrueckii* subsp. *bulgaricus*. ^d^ One subject in the control group withdrew from the study before receiving study product and was not included in the ITT analysis. ^e^ A total of 21 subjects were not included in the PP analysis due to protocol deviations, which included withdrawals (8 in probiotic group and 13 in control group). ^f^ A total of 100 subjects were not included in the PP analysis due to major protocol deviations, which included withdrawals (57 in probiotic group and 43 in control group). ^g^ The intervention period included a 3-wk training period and a 5-day combat course. ^h^ It was NR whether any subjects withdrew from the study; thus, it was assumed that all subjects completed the study as the number of subjects randomized was identical to the number of subjects analyzed. ^i^ A total of 19 subjects withdrew prior to the start of product consumption and were not included in the ITT analysis. ^j^ A total of 208 subjects were not included in the PP analysis due to one or more protocol deviations, which included withdrawals (107 in probiotic group and 101 in control group).

**Table 3:** Assessment of additional potential confounders pertinent to studies wherein common infectious diseases (CIDs) were assessed.

Additional Potential Confounders Considered in Scoring Criteria #6, #10, and #11 of the NIH Quality Appraisal Tool	Children		Adults			Elderly				Accounted For
	Merenstein et al. [24]	Prodeus et al. [2]	Guillemard et al. [39]	Pereg et al. [49]	Tiollier et al. [50]	Boge et al. ([40], Pilot and Confirmatory Studies) Pilot Study	Boge et al. ([40], Pilot and Confirmatory Studies) Confirmatory Study	Guillemard et al. [42]	Turchet et al. [41]	
6. Were the groups similar at baseline on important characteristics that could affect outcomes (e.g., demographics, risk factors, and co-morbid conditions)? ^a^	Partially	✓	✓	NR	NR	✓	✓	✓	Partially ^b^	5/9
(1) Presence of CIDs at baseline	✓	✓	✓	NR	NR	✓	✓	✓	✓	7/9
(2) Influenza or rotavirus vaccination status at baseline	NR	✓	✓	NR	NR	✓	✓	✓	✓	6/9
(3) Medication/supplement use at baseline (e.g., proton pump inhibitors)	✓	✓	✓	NR	NR	✓	✓	✓	✓	7/9
10. Were other interventions avoided or similar in the groups (e.g., similar background treatments)? ^c^	✓	✓	✓	NR	Partially	Partially	Partially	✓	NR/No	4/9
(1) Use of rescue medications/supplements during study (e.g., for colds, flu, or diarrhea)	✓	✓	✓	NR	NR	NR	NR	✓	NR	4/9
(2) Consumption of other probiotics during study	✓	✓	✓	NR	✓	✓	✓	✓	No	7/9
11. Were outcomes assessed using valid and reliable measures, implemented consistently across all study participants? ^d^	Partially	✓	Partially	Partially	Partially	NR	NR	✓	Partially	2/9
(A) Incidence of CIDs	Partially	✓	✓	Partially	Partially	NR	NR	✓	✓	4/9
(1) Diagnosed by a physician/health professional	No	✓	✓	No	No	NR	NR	✓	✓	4/9
(2) If not diagnosed, type of symptoms listed (e.g., sneezing or runny nose)	✓	NA	NA	✓	✓	NR	NR	NA	NA	3/5
(3) If not diagnosed, number and duration of symptoms used to define a CID episode (e.g., must have at least two symptoms within two consecutive days)	NR	NA	NA	NR	✓	NR	NR	NA	NA	1/5
(B) Duration of CIDs: how duration was determined (e.g., first to the last day of symptoms)	Not assessed	✓	NR	NR	NR	NR	NR	✓	NR	2/8
(C) Severity of CIDs: how severity was determined (e.g., scoring system: mild, moderate, or severe)	Not assessed	✓	✓	Not assessed	✓	NR	NR	✓	NR	4/7

✓: yes; CID: common infectious disease; NIH: National Institutes of Health; NR: not reported. ^a^ In addition to general demographic characteristics (e.g., age and gender), the following characteristics at baseline were considered: 1) the presence of CIDs; 2) influenza or rotavirus vaccination status; and 3) medication/supplement use (e.g., proton pump inhibitors). ^b^ The following was reported in the study: “The mean age of the treatment group was 67.1 ± 6.0 years, and for the control group 69.3 ± 5.6 [years]. Although this difference was statistically significant, it is not considered clinically significant.” However, age is considered an important confounder particularly in this population group of elderly subjects. The *p*-value for the significant difference in age was not reported in the study. ^c^ The following other interventions/background treatments during the study were considered: 1) the use of rescue medications/supplements (e.g., for colds, flu, or diarrhea); and 2) the consumption of other probiotics. ^d^ The following characteristics related to the measures used to assess the outcomes were considered: A) the incidence of CIDs: 1) diagnosed by a physician/health professional; 2) if not diagnosed by a physician/health professional, the type of symptoms listed (e.g., sneezing or runny nose); and 3) if not diagnosed by a physician/health professional, the number and duration of symptoms used to define a CID episode (e.g., must have at least two symptoms within two consecutive days); B) the duration of CIDs: how duration was determined (e.g., first to the last day of symptoms); C) the severity of CIDs: how severity was determined (e.g., scoring system: mild, moderate, or severe).

**Table 4:** Map of outcomes and metrics assessed across studies.

Outcome/Metric Assessed	Children		Adults			Elderly				Number of Studies in which Metric was Assessed
	Merenstein et al. [24]	Prodeus et al. [2]	Guillemard et al. [39]	Pereg et al. [49]	Tiollier et al. [50]	Boge et al. ([40], Pilot and Confirmatory Studies) Pilot Study	Boge et al. ([40], Pilot and Confirmatory Studies) Confirmatory Study	Guillemard et al. [42]	Turchet et al. [41]	
**Incidence**										
Relative risk	✓	✓	✓					✓		4 ^a^
Mean CIDs per subject			✓					✓ ^b^		2 ^a^
Subjects with ≥1 CID			✓					✓	✓ ^c^	3 ^a^
Subjects with diarrhea				✓						1
Subjects who vomited amongst subjects with diarrhea				✓						1
Subjects with abdominal pain amongst subjects with diarrhea				✓						1
"Mean maximal number of watery stools/day"				✓						1
**Duration**										
Mean cumulative days of CIDs amongst subjects with CIDs		✓						✓	✓ ^d^	3 ^a^
Mean cumulative days of CIDs amongst all subjects			✓							1
Mean days per CID episode amongst subjects with CIDs		✓	✓					✓		3 ^a^
“Mean duration of diarrhea (days)”				✓						1
**Severity**										
Cumulative number of CIDs categorized as “mild”		✓								1
Cumulative number of CIDs categorized as “moderate”		✓								1
“Severity of CID”			✓ ^e^							1
Cumulative number of CIDs categorized as “severe” amongst subjects with CIDs			✓					✓		2 ^a^
"Severity of symptoms (mild, moderate, severe)"					✓ ^e^					1
"Severity of CID or influenza illnesses"						✓^e^	✓ ^e^			2
"Intensity"									✓^e^	1

✓: outcome/metric assessed in study; CID: common infectious disease. ^a^ Metric for which a meta-analysis was conducted. ^b^ Metric was reported as "mean rate" of CIDs across the "whole population." ^c^ Metric was reported as “subjects with winter pathologies." ^d^ Metric was reported as mean “duration (days) of the pathologies” amongst subjects with winter pathologies. ^e^ Numerical results were not reported.

**Table 5:** Combined effects of a fermented dairy drink containing *Lacticaseibacillus paracasei* subsp. *paracasei* CNCM I-1518 and the standard yogurt cultures on common infectious diseases (CIDs).

Analysis	Metric	Studies	Study Quality	Meta-Analysis Results		Heterogeneity		Publication Bias
				Combined Effect (95% CI)	*P*-Value	I^2^	*p*-Value	
Incidence	RR	*n* = 4 (*n* = 2 children; *n* = 1 adults; *n* = 1 elderly)	*n* = 3 good *n* = 1 poor	RR = 0.91 (0.82, 1.01)	0.082	0.00	0.405	No
	Mean number of CIDs per subject	*n* = 2 (*n* = 1 adults; *n* = 1 elderly)	*n* = 2 good	−0.09 (−0.15, −0.04)	0.001	0.00	0.567	Insufficient data
	Subjects with ≥1 CID	*n* = 3 (*n* = 1 adults; *n* = 2 elderly)	*n* = 2 good *n* = 1 poor	OR = 0.81 (0.66, 0.98)	0.029	14.84	0.309	No
Duration	Mean cumulative days of CIDs amongst subjects with CIDs	*n* = 3 (*n* = 1 children; *n* = 2 elderly)	*n* = 2 good *n* = 1 poor	−1.31 (−2.89, 0.28)	0.106	55.85	0.079	Yes ^a^
	Mean days per CID episode amongst subjects with CIDs	*n* = 3 (*n* = 1 children; *n* = 1 adults; *n* = 1 elderly)	*n* = 3 good	−0.29 (−1.55, 0.97)	0.653	76.28	0.015	No
Severity	Cumulative number of CIDs categorized as “severe” amongst subjects with CIDs	*n* = 2 (*n* = 1 adults; *n* = 1 elderly)	*n* = 2 good	OR = 0.99 (0.54, 1.81)	0.968	0.00	0.903	Insufficient data

CI: confidence interval; CID: common infectious disease; n: number; OR: odds ratio; RR: relative risk. ^a^ According to the trim and fill method of Duval and Tweedie [46], one study was found to be missing to the right of the combined effect. With this study imputed, the mean cumulative days of infection in subjects with CIDs was −0.86 (95% CI: −2.45, 0.72) days.

## Supplementary Materials

The following is available online at https://www.mdpi.com/2072-6643/12/11/3443/s1, Table S1: Study quality according to the NIH quality appraisal tool.
