# Supplementary material for: Effects of a Fermented Dairy Drink Containing Lacticaseibacillus paracasei subsp. paracasei CNCM I-1518 (Lactobacillus casei CNCM I-1518) and the Standard Yogurt Cultures on the Incidence, Duration, and Severity of Common Infectious Diseases: A Systematic Review and Meta-Analysis of Randomized Controlled Trials
Source: Nutrients. 2020 Nov 10;12(11):3443. doi: 10.3390/nu12113443 (PMC7698120; doi:10.3390/nu12113443)
Supplement: Supplementary file 1 [file nutrients-12-03443-s001.pdf]

Supplementary Table S1. Study quality according to the National Institutes of Health (NIH) quality appraisal tool

Quality Criteria	Children		Adults			Elderly			
	**Merenstein et al. [24]**	**Prodeus et al. [2]**	**Guillemard et al. [39]**	**Pereg et al. [49]**	**Tiollier et al. [50]**	**Boge et al. [40] Pilot Study**	**Boge et al. [40] Confirmatory Study**	**Guillemard et al. [42]**	**Turchet et al. [41]**
1. Was the study described as randomized, a randomized trial, a randomized clinical trial, or an RCT?	✓	✓	✓	✓	✓	✓	✓	✓	✓
2. Was the method of randomization adequate (i.e., use of randomly generated assignment)?	✓[1]	✓[2]	No[3]	NR	NR	✓[4]	✓[4]	No[5]	NR
3. Was the treatment allocation concealed (so that assignments could not be predicted)?	✓[1]	✓[2]	✓[3]	NR	NR	✓[4]	✓[4]	✓[5]	NR
4. Were study participants and providers blinded to treatment group assignment?	✓[6]	✓[7]	✓[8]	No[9]	✓[10]	✓[11]	✓[11]	✓[12]	No[13]
5. Were the people assessing the outcomes blinded to the participants' group assignments?	✓[6]	✓[7]	✓[8]	No[9]	NR[10]	✓[11]	✓[11]	✓[12]	NR
6. Were the groups similar at baseline on important characteristics that could affect outcomes (e.g., demographics, risk factors, co-morbid conditions)?	Partially[14]	✓[15]	✓[16]	NR[17]	NR[18]	✓[19]	✓[19]	✓[20]	Partially[21]
7. Was the overall drop-out rate from the study at endpoint 20% or lower of the number allocated to treatment?	✓[22]	✓[23]	✓[24]	✓[25]	✓[26]	✓[27]	✓[28]	✓[29]	✓[30]
8. Was the differential drop-out rate (between treatment groups) at endpoint 15 percentage points or lower?	✓[31]	✓[32]	✓[33]	✓[34]	✓[26]	✓[35]	NR[36]	NR[37]	✓[38]

Quality Criteria	Children		Adults			Elderly			
	**Merenstein et al. [24]**	**Prodeus et al. [2]**	**Guillemard et al. [39]**	**Pereg et al. [49]**	**Tiollier et al. [50]**	**Boge et al. [40] Pilot Study**	**Boge et al. [40] Confirmatory Study**	**Guillemard et al. [42]**	**Turchet et al. [41]**
9. Was there high adherence to the intervention protocols for each treatment group?	✓[39]	✓[40]	✓[41]	✓[42]	✓[43]	✓[44]	✓[45]	✓[46]	NR[47]
10. Were other interventions avoided or similar in the groups (e.g., similar background treatments)?	✓[48]	✓[49]	✓[50]	NR[51]	Partially[52]	Partially[53]	Partially[53]	✓[54]	NR/No[55]
11. Were outcomes assessed using valid and reliable measures, implemented consistently across all study participants?	Partially[56]	✓[57]	Partially[58]	Partially[59]	Partially [60]	NR[61]	NR[61]	✓[62]	Partially[63]
12. Did the authors report that the sample size was sufficiently large to be able to detect a difference in the main outcome between groups with at least 80% power?	✓[64]	✓[65]	✓[66]	NR	NR	NR[67]	No[68]	✓[69]	NR
13. Were outcomes reported or subgroups analyzed prespecified (i.e., identified before analyses were conducted)?	✓	✓[70]	No[71]	✓	✓	✓	✓	✓	No[72]
14. Were all randomized participants analyzed in the group to which they were originally assigned, i.e., did they use an ITT analysis?	✓[73]	✓[74]	✓[75]	No[76]	✓[77]	✓[78]	✓[78]	✓[79]	✓[80]
Quality Rating (Good, Fair, or Poor)[81]	Poor[82]	Good	Good	Poor	Poor	Fair	Poor[83]	Good	Poor

AGGIR: Autonomy Gerontology Iso-Resources Group; CID: common infectious disease; GITI: gastrointestinal tract infection; ITT: intention to treat; LRTI: lower respiratory tract infection; NA: not applicable; NIH: National Institutes of Health; NR: not reported; PP: per protocol; RCT: randomized controlled trial; URTI: upper respiratory tract infection.

[1] "The randomization scheme was generated using SAS software by data managers, who had no participant contact… Once eligibility criteria were met, the participant was randomized to one of the two groups, study identification was generated and a number from 0 to 9 was assigned… Through masking and use of 10 different numbers, 0 through 9, it was impossible for research personnel to adjust randomization or deduce what group participants were assigned."

[2] "To avoid selection bias, the randomization scheme was generated by data managers in line with the Cochrane guidelines… using Statistical Analysis Software… The randomization list was kept confidential at the sponsor's premises and remained confidential with the exception to those involved in the production of the study products and for biostatistical managers (after the first part of data locking was performed)."

[3] Although it was reported that "volunteers were randomly allocated to verum or control group using an individual randomization number (study product allocation concealed) and were included sequentially according with the randomization list," the method of randomization was not specified.

[4] "Randomisation was conducted by random number allocation prepared prior to the start of the study; this was centralised, confidential/blind and supervised by an external independent contractor. Randomisation was stratified by study centre and AGGIR score, where each participant was allocated to either of the 2 study groups using a randomisation number from the blinded list."

[5] Although it was reported that "volunteers were assigned to study groups using an individual randomisation number (study product allocation concealed) and were included sequentially in accordance with the randomisation list, which was stratified by centre," the method of randomization was not specified.

[6] The study was reported as double-blinded. In addition, the "statisticians masked to the group allocation conducted statistical analyses. Furthermore, all research personnel were masked while examining initial data."

[7] The study was reported as double-blinded. In addition, the study ensured "the blinding of both study participants and key study personnel including the outcome assessors. Code-breaking systems were available in case of occurrence of a serious adverse event for which the medical personnel needed to be aware of what the participant received (active or control product). Raw data were also blinded during the blind review. The code was broken after the database was locked."

[8] The study was reported as double-blinded. In addition, "all biological analyses of blood parameters and pathogens were performed blind with standard procedures, in a subpopulation of 200 volunteers."

[9] The study was reported as single-blinded. Given that the study products were supplied in a similar packaging, it was assumed that the subjects were blinded.

[10] The subjects were blinded. Although it is unclear whether the providers or outcome assessors were blinded, the study was reported as double-blinded.

[11] The study was double-blinded. In addition, the specific serum antibody titres against the 3 influenza vaccine strains, which was the primary outcome measured, were assessed by a hemagglutination inhibition assay conducted in blind by the central laboratory Texcell SA (Evry, France) and the National Influenza Centre (Northern France).

[12] The study was double-blinded. The authors reported that "all the analytical dosages in the study were performed in blind"; thus, it was assumed that this referred to the blinding of the outcome assessors.

[13] "This was a unicentric, randomised, stratified, open pilot study. Subjects were randomised to the treatment group… or control group (no study product)…"

[14] In addition to baseline demographics, the following baseline characteristics were considered:

1) Presence of CIDs at baseline: Yes; "exclusion criteria were… active respiratory or gastrointestinal infection, or chronic disease."

2) Influenza or rotavirus vaccination status at baseline: NR

3) Medications/supplements at baseline (e.g., proton pump inhibitors): Yes; "exclusion criteria were taking any regular medicines at initiation of study."

[15] In addition to baseline demographics, the following baseline characteristics were considered:

1) Presence of CIDs at baseline: Yes; "volunteers were… free from respiratory and gastrointestinal tract symptoms… Volunteers having experienced an infectious disease 7 days before study entry were excluded." There were no significant differences between groups at baseline with respect to the number of subjects with any infectious diseases during the previous month and year.

2) Influenza or rotavirus vaccination status at baseline: Yes; "subjects were excluded from participation if: current or recent systemic or topical treatment likely to interfere with the study parameters (antibiotics, antiseptics, corticosteroids, vaccines, antifungal, or antihistaminic agents)…" There were no significant differences between groups at baseline with respect to the number of subjects with vaccinations during the previous year and the mean number of days of delay in flu vaccination before selection time.

3) Medications/supplements at baseline (e.g., proton pump inhibitors): Yes; see above.

[16] In addition to baseline demographics, the following baseline characteristics were considered:

1) Presence of CIDs at baseline: Yes; "inclusion criteria were as follows… no symptoms of CIDs during the 2 weeks before product consumption… Exclusion criteria included… severe respiratory allergy…" No significant difference between groups at baseline with respect to history of infectious disease during the last year. As part of the statistical analyses, "for the primary criteria, history of CIDs during the last month and study duration were also taken into account."

2) Influenza or rotavirus vaccination status at baseline: Yes; no significant difference between groups at baseline with respect to vaccination against influenza.

3) Medications/supplements at baseline (e.g., proton pump inhibitors): Yes; "exclusion criteria included… chronic disease requiring antibiotics/antiseptics/anti-inflammatory medications…" No significant difference between groups at baseline with respect to "treatment at inclusions."

[17] In addition to baseline demographics, the following baseline characteristics were considered:

1) Presence of CIDs at baseline: NR

2) Influenza or rotavirus vaccination status at baseline: NR

3) Medications/supplements at baseline (e.g., proton pump inhibitors): NR

[18] In addition to baseline demographics, the following baseline characteristics were considered:

1) Presence of CIDs at baseline: NR

2) Influenza or rotavirus vaccination status at baseline: NR

3) Medications/supplements at baseline (e.g., proton pump inhibitors): NR

[19] In addition to baseline demographics, the following baseline characteristics were considered:

1) Presence of CIDs at baseline: Yes; there were no differences between the two groups in terms of baseline demographics, medical history or current disease (Table 2)."

2) Influenza or rotavirus vaccination status at baseline: Yes; "volunteers already protected against the H3N2 viral strain contained in the concerned vaccine were excluded (antibody titre superior or equal to 40)."

3) Medications/supplements at baseline (e.g., proton pump inhibitors): Yes; "volunteers had no pathology requiring antibiotic treatment."

[20] In addition to baseline demographics, the following baseline characteristics were considered:

1) Presence of CIDs at baseline: Yes; "exclusion criteria included… any current or past severe respiratory, gastrointestinal or metabolic pathology… any infection in the last 14 days…" There was no significant difference between groups at baseline with respect to CIDs in the last month or year.

2) Influenza or rotavirus vaccination status at baseline: Yes; "vaccination against the influenza virus at least 14 days before inclusion…"

3) Medications/supplements at baseline (e.g., proton pump inhibitors): Yes; "the restriction implied the exclusion of… over-the counter medication containing probiotics, vitamins, minerals, or other nutrients… Exclusion criteria included… currently receiving or having received in the 4 last weeks; drugs likely to interfere with evaluation of the study parameters, including antibiotics, intestinal or respiratory antiseptics, anti-fungal (except topical), corticoids, vaccines (except influenza vaccine), anti-histaminic molecules, non-corticoid anti-inflammatory substances (except aspirin or equivalent at doses preventing from aggregation of platelets or blood clotting) and immunosuppressant treatment."

[21] "The mean age of the treatment group was 67.1 ± 6.0 years, and for the control group 69.3 ± 5.6 [years]. Although this difference was statistically significant, it is not considered clinically significant." However, age is considered an important confounder particularly in this population group of elderly subjects. The P-value for the significant difference in age was not reported in the study. In addition to baseline demographics, the following baseline characteristics were considered:

1) Presence of CIDs at baseline: Yes; "people with chronic infection requiring antibiotic therapy more than three times per year… were excluded."

2) Influenza or rotavirus vaccination status at baseline: Yes; "82% of subjects had been vaccinated 3 months before the study against influenza. The vaccination rate was similar in the two groups."

3) Medications/supplements at baseline (e.g., proton pump inhibitors): Yes; "people with chronic infection requiring antibiotic therapy more than three times per year… chronic anti-inflammatory medication (steroidal or non-steroidal), antibiotic therapy in the previous 3 weeks… were excluded."

[22] It is unclear whether subjects who "missed >3 consecutive or 4 total follow-ups" dropped out of the study; thus, as a conservative estimate, these subjects were considered drop-outs. Based on the number of subjects withdrawn or who missed >3 consecutive or 4 total follow-ups, the overall drop-out rate was 49/638 (7.7%).

[23] The study authors reported that 16 subjects dropped out of the study. However, based on the information reported in the study, it is unclear whether the 3 subjects with major protocol deviations related to compliance who were withdrawn prematurely from the study were already included amongst the 16 subjects who dropped out of the study. Thus, based on a conservative estimate that these 3 subjects were in addition to the 16 drop-outs, the overall drop-out rate was 19/600 (3.2%).

[24] The overall drop-out rate was 38/1000 (3.8%).

[25] The overall drop-out rate was 39/541 (7.2%).

[26] It appears that all subjects completed the study; thus, the overall drop-out and differential rates were 0%.

[27] The overall drop-out rate was 11/86 (12.8%).

[28] The overall drop-out rate was 46/241 (19.1%).

[29] The overall drop-out rate was 46/1,072 (4.3%).

[30] The authors reported that "only two subjects were prematurely withdrawn from the treatment group;" however, it is unclear whether any subjects from the control group were withdrawn from the study. Based on the assumption that the 2 subjects from the treatment group were the only drop-outs in the study, the overall drop-out rate was 2/360 (0.6%).

[31] It is unclear whether subjects who "missed >3 consecutive or 4 total follow-ups" dropped out of the study; thus, as a conservative estimate, these subjects were considered drop-outs. Based on the number of subjects withdrawn or who missed >3 consecutive or 4 total follow-ups, the drop-out rates in the active and control groups were 19/314 (6.1%) and 30/324 (9.3%), respectively; the differential drop-out rate was 3.2%.

[32] The study authors reported that 16 subjects dropped out of the study. However, based on the information reported in the study, it is unclear whether the 3 subjects with major protocol deviations related to compliance who were withdrawn prematurely from the study were already included amongst the 16 drop-outs, and unclear to which group the subjects belonged. Thus, not including the 3 aforementioned subjects, the drop-out rates in the active and control groups were 6/300 (2.0%) and 10/299 (3.3%), respectively; the differential drop-out rate was 1.3%. "There were only few missing data because of premature withdrawal, and they were balanced in numbers across the 2 groups with similar reasons for missing data. This makes reasons for missing data unlikely to be related to true outcome and unlikely to have a clinically relevant impact on the intervention effect estimate indicating low risk of attrition bias according to the Cochrane Collaboration."

[33] The drop-out rates in the active and control groups were 22/500 (4.4%) and 16/500 (3.2%), respectively; the differential drop-out rate was 1.2%.

[34] The drop-out rates in the active and control groups were 21/275 (7.6%) and 18/266 (6.8%), respectively; the differential drop-out rate was 0.8%.

[35] The drop-out rates in the active and control groups were 3/44 (6.8%) and 8/42 (19.0%), respectively; the differential drop-out rate was 12.2%.

[36] The group allocation was NR for 19 subjects who were randomized but withdrawn from the study prior to the consumption of the investigational product; thus, the differential drop-out rate could not be calculated based on the information reported in the study.

[37] The drop-out rates in the active and control groups were NR; thus, the differential drop-out rate could not be determined based on the information reported in the study.

[38] The authors reported that "only two subjects were prematurely withdrawn from the treatment group;" however, it is unclear whether any subjects from the control group were withdrawn from the study. Based on the assumption that the 2 subjects from the treatment group were the only drop-outs in the study, the differential drop-out rate was 0.6%.

[39] Although it appeared that compliance with product consumption was high, the authors reported that "the number of drinks consumed per week differed between the active (6.5 drinks per week) and control (6.1 drinks per week) groups (P=0.004)."

[40] The compliance rates for the active and control groups were 98.6 and 98.0%, respectively.

[41] "Compliance was not significantly different between groups (p=0.212), and 97.5 (0.1)% (mean (SD)) of the provided bottles of product were consumed in the whole population."

[42] Although the compliance rates for the active and control groups were NR, the subjects were military recruits residing in a single camp, and the study products "were handed personally to each participant and were consumed immediately, allowing close monitoring and ensuring high compliance."

[43] The compliance rates for the active and control groups were 90.9 and 93.5%, respectively. "The compliance for the test product was… not different between groups… P=0.84…).

[44] The compliance rates for the active and control groups were 97.0 and 98.5%, respectively.

[45] The overall compliance rate was "96.3% with no significant difference between groups."

[46] The compliance rate was 100% for both the active and control groups.

[47] The compliance rates for the active and control groups were NR. Importantly, the prescribed dose of fermented milk (one 100 mL bottle, twice daily) changed during the study for a portion of the subjects. "In the treatment group, 19 men (31.7%) and 26 women (21.7%) experienced dyspepsia (bloating, meteorism, nausea) during the study. For this reason the intake of the treatment was reduced from two bottles to one bottle per day."

[48] The following interventions/background treatments were considered:

1) Use of rescue medications/supplements (e.g., for colds, flu, diarrhea) during study: Yes; although there were significant differences between groups in medication use during the study (which the authors believed to be not clinically significant), the results were in favour of a beneficial effect of the fermented dairy drink. "In the active group, the mean number of days a medicine used was 3.02 days versus 3.32 days in the control group (P<0.0001). Furthermore, there was also a significant statistical difference in antibiotics (n=58 in intervention and n=69 for control, P=0.002) and anti-inflammatory (n=77 in intervention and n=97 in control, P=0.03) drug usage in the control group compared with the active group, when used as a covariate in the primary analysis model. However, the absolute numbers are not large and we believe not clinically significant."

2) Consumption of probiotics during study: Yes; "participants were also excluded for consuming other probiotic foods or supplements."

[49] The following interventions/background treatments were considered:

1) Use of rescue medications/supplements (e.g., for colds, flu, diarrhea) during study: Yes; "there was no significant difference between the groups for the number of subjects who took medications for CID, neither for the number of medications nor the duration of intake, whatever the study period being investigated."

2) Consumption of probiotics during study: Yes; "during the study phase… subjects were asked to abstain from vitamin or probiotics supplementation and any product containing live ferments: probiotic drinks, kefir, and fresh yoghurts. Other dairy products were allowed that do not contain live bacteria (or low rate): milk, soft and hard cheeses, sour cream, cream, butter, ice cream, and pasteurized fermented dairies… Caregivers were required to complete the diary by recording… consumption of nonallowed products (if yes, by reporting the name and ingested amount)."

[50] The following interventions/background treatments were considered:

1) Use of rescue medications/supplements (e.g., for colds, flu, diarrhea) during study: Yes; "no difference was seen between groups in CID-associated total medication for prescribed and self-medications taken together (a somewhat higher occurrence of prescribed medication in the verum group for the subset of URTI/rhinopharyngitis did not sum up to a different total medication in this subset also)."

2) Consumption of probiotics during study: Yes; "subjects were asked to avoid consumption of other fermented dairy products with probiotics, yogurts, and over-the-counter medications containing probiotics or vitamins, from 2 weeks before study product consumption to the end of the study."

[51] The following interventions/background treatments were considered:

1) Use of rescue medications/supplements (e.g., for colds, flu, diarrhea) during study: NR

2) Consumption of probiotics during study: NR

[52] The following interventions/background treatments were considered:

1) Use of rescue medications/supplements (e.g., for colds, flu, diarrhea) during study: NR

2) Consumption of probiotics during study: Yes; "the ration did not contain any fermented dairy products… During the 3 weeks preceding the beginning of the training, the subjects were allowed to eat no more than one fermented dairy product per day… During the period of supplementation, they were asked to avoid other fermented dairy products, which was easy to control since all of the food was provided by the commandment."

[53] The following interventions/background treatments were considered:

1) Use of rescue medications/supplements (e.g., for colds, flu, diarrhea) during study: NR

2) Consumption of probiotics during study: Yes; "in both studies, volunteers were instructed to avoid taking any other fermented dairy products or yoghurts from screening until the end of the product consumption period, in order to minimise interference with the study results."

[54] The following interventions/background treatments were considered:

1) Use of rescue medications/supplements (e.g., for colds, flu, diarrhea) during study: Yes; "there was no difference between the two groups of participants regarding… CID-associated medication (prescribed and/or auto-administered)… the proportion of volunteers receiving at least one prescription of medication was 67.5 [versus] 64.2% in the fermented and the control product group, respectively."

2) Consumption of probiotics during study: Yes; "dietary restriction during the 2 weeks preceding the product consumption phase and throughout the study. The restriction implied the exclusion of fermented dairy products with probiotics other than those used in the study, yoghurts and over-the counter medication containing probiotics, vitamins, minerals, or other nutrients."

[55] The following interventions/background treatments were considered:

1) Use of rescue medications/supplements (e.g., for colds, flu, diarrhea) during study: NR

2) Consumption of probiotics during study: No; "all subjects could ingest no more than two additional servings of other fermented dairy products per week."

[56] The following measures used to assess the outcomes were considered:

1) Incidence of CIDs: Partially

a) Diagnosed by a physician/health professional: No; "CID was categorized based on the reported health-related symptoms that parents relayed on a weekly basis to the research personnel… Diarrhea was not clinically defined, but parent reported."

b) If not diagnosed by a physician/health professional, type of symptoms listed (e.g., sneezing, runny nose): Yes; "CID was separated into three categories of infections, *a priori* to review of data: URTIs, which included ear infections, sinusitis, streptococcal pharyngitis, non-strep pharyngitis, nasal discharge, and laryngitis; LRTIs, which included pneumonia, influenza, coughs, and breathing problems; and GITIs, which included gastroenteritis, diarrhea, nausea, and vomiting."

c) If not diagnosed by a physician/health professional, number and duration of symptoms used to define a CID episode (e.g., must have at least 2 symptoms within 2 consecutive days): NR; although it was reported that "the overall CID at each follow-up visit (with period covering 1 week) could be ≤3", it was unclear whether this information was used to define an episode of CID.

2) Duration of CIDs: Not assessed

a) How duration was determined (e.g., first to the last day of symptoms): NA

3) Severity of CIDs: Not assessed

a) How it was determined (e.g., scoring system: mild, moderate, severe): NA

[57] The following measures used to assess the outcomes were considered:

1) Incidence of CIDs: Yes

a) Diagnosed by a physician/health professional: Yes; "caregivers were required to complete the diary by recording… occurrence of illness… report of the category and type of infection such as URTI (rhinopharyngitis, sore throat, sinusitis, and otitis), LRTI (acute bronchitis, pneumonia, flu, or flu-like illness), and GITI (gastroenteritis)…" In addition to the regularly scheduled clinic visits, "additional visits were conducted when any CID occurred… Other visits resulting from the occurrence of clinical symptoms related to CIDs were conducted 2 to 4 days following the start of such symptom. The diagnosis of CID was assessed by the physician, and data on the type and severity of the infection, prescribed medication, and subject's temperature were recorded. For each CID, the start date, end date, and global severity were recorded, and the number of days of sick leave from day-care and of parental absence from work was noted from the personal diary. A clinical examination was also performed at each additional visit, and biological samples were collected for pathogens analysis according to the CID type (nasal discharge, and/or throat swabs, and/or stools)."

b) If not diagnosed by a physician/health professional, type of symptoms listed (e.g., sneezing, runny nose): NA

c) If not diagnosed by a physician/health professional, number and duration of symptoms used to define a CID episode (e.g., must have at least 2 symptoms within 2 consecutive days): NA

2) Duration of CIDs: Yes

a) How duration was determined (e.g., first to the last day of symptoms): Yes; the duration of CIDs was calculated based on the start and end date of each CID noted in the subject's personal diary.

3) Severity of CIDs: Yes

a) How it was determined (e.g., scoring system: mild, moderate, severe): Yes; "caregivers were required to complete the diary by recording… occurrence of illness (if yes, record global severity with 1 mild, 2 moderate, and 3 severe)…" In addition to the regularly scheduled clinic visits, "additional visits were conducted when any CID occurred… Other visits resulting from the occurrence of clinical symptoms related to CIDs were conducted 2 to 4 days following the start of such symptom. The diagnosis of CID was assessed by the physician, and data on the type and severity of the infection, prescribed medication, and subject's temperature were recorded. For each CID, the start date, end date, and global severity were recorded, and the number of days of sick leave from day-care and of parental absence from work was noted from the personal diary."

[58] The following measures used to assess the outcomes were considered:

1) Incidence of CIDs: Yes

a) Diagnosed by a physician/health professional: Yes; "in case of disease, volunteers recorded daily in a diary any of their symptoms, body temperature, and medications during the whole period of disease, and they attended an additional evaluation 3±(1) days after the onset of symptoms. The investigator clinically assessed the diagnosis of CID and reported the start date (first day of symptoms) and end date (last day of symptoms). A CID diagnosed during the disease was termed 'regular reported CID'. A CID diagnosed on the basis of symptoms reported by a volunteer after the end of the episode at the following visit was defined as 'delayed reported CID'… The occurrence and amount of specific pathogens were assessed in biological samples in cases of CID. For URTIs, influenza virus A and B, coronavirus, rhinovirus, and enterovirus were assessed in the upper airways. In the specific case of sore throat and LRTIs, Streptococcus pneumoniae, Staphylococcus aureus, Streptococcus pyogenes (b-hemolytic group A), and Haemophilus influenzae were analyzed. For GITIs, stools were assessed for Salmonella spp, Shigella spp, Campylobacter spp, and Yersinia enterolitica."

b) If not diagnosed by a physician/health professional, type of symptoms listed (e.g., sneezing, runny nose): NA

c) If not diagnosed by a physician/health professional, number and duration of symptoms used to define a CID episode (e.g., must have at least 2 symptoms within 2 consecutive days): NA

2) Duration of CIDs: NR

a) How duration was determined (e.g., first to the last day of symptoms): NR

3) Severity of CIDs: Yes

a) How it was determined (e.g., scoring system: mild, moderate, severe): Yes; "the severity of CIDs was assessed with a 3-point scale (1, mild; 2, moderate; 3, severe), based on the degree of interference with the subject's daily activity, as evaluated by the investigator."

[59] The following measures used to assess the outcomes were considered:

1) Incidence of CIDs: Partially

a) Diagnosed by a physician/health professional: No; "participants were asked to report any incidence of diarrhea, starting the second week of the study… Upon reporting diarrhea, the participants answered a questionnaire and were followed for clinical symptoms and signs as well as the duration of diarrheal episode."

b) If not diagnosed by a physician/health professional, type of symptoms listed (e.g., sneezing, runny nose): Yes; "diarrhea was defined as the passage of 3 or more loose stools in a 24-hour period."

c) If not diagnosed by a physician/health professional, number and duration of symptoms used to define a CID episode (e.g., must have at least 2 symptoms within 2 consecutive days): NR

2) Duration of CIDs: NR

a) How duration was determined (e.g., first to the last day of symptoms): NR

3) Severity of CIDs: Not assessed

a) How it was determined (e.g., scoring system: mild, moderate, severe): NA

[60] The following measures used to assess the outcomes were considered:

1) Incidence of CIDs: Partially

a) Diagnosed by a physician/health professional: No; "cadets were requested to report daily, via a logbook, their signs and symptoms of infection, giving detailed Information on [respiratory tract infections]. Also, as of the study, medical examinations were carried out on four occasions: before and at the end of the 3-week training, at the end of the 5-day course and after 1 week of recovery. Based on the collected data and on the examinations, the medical officers filled in a standardized log sheet giving details on the symptoms (rhinopharyngitis [defined as inflammation of the mucous membranes of the nasal and the pharyngeal cavities), tonsillitis, sinusitis, otitis, bronchitis, pneumonia, and asthma), the severity of the symptoms (mild, moderate, or severe), and the duration of the symptoms so far."

b) If not diagnosed by a physician/health professional, type of symptoms listed (e.g., sneezing, runny nose): Yes; see above.

c) If not diagnosed by a physician/health professional, number and duration of symptoms used to define a CID episode (e.g., must have at least 2 symptoms within 2 consecutive days): Yes; "more than one symptom could be diagnosed on the same day for any one subject. [A respiratory tract infection] was recorded when subjects reported infectious symptoms on at least 2 consecutive days."

2) Duration of CIDs: NR

a) How duration was determined (e.g., first to the last day of symptoms): NR

3) Severity of CIDs: Yes

a) How it was determined (e.g., scoring system: mild, moderate, severe): Yes; see above.

[61] The following measures used to assess the outcomes were considered:

1) Incidence of CIDs: NR; the incidence of CIDs was assessed as part of the adverse event reporting in the study, and details related to the diagnosis of and symptoms used to define CIDs were NR.

a) Diagnosed by a physician/health professional: NR

b) If not diagnosed by a physician/health professional, type of symptoms listed (e.g., sneezing, runny nose): NR

c) If not diagnosed by a physician/health professional, number and duration of symptoms used to define a CID episode (e.g., must have at least 2 symptoms within 2 consecutive days): NR

2) Duration of CIDs: NR

a) How duration was determined (e.g., first to the last day of symptoms): NR; it was only reported in the discussion that "…no significant difference between the probiotic and control groups could be evidenced for the severity, duration, or incidence of CID or influenza illnesses."

3) Severity of CIDs: NR; it was only reported in the discussion that "…no significant difference between the probiotic and control groups could be evidenced for the severity, duration, or incidence of CID or influenza illnesses."

a) How it was determined (e.g., scoring system: mild, moderate, severe): NR

[62] The following measures used to assess the outcomes were considered:

1) Incidence of CIDs: Yes

a) Diagnosed by a physician/health professional: Yes; in addition to the regularly scheduled clinic visits, "volunteers attended an additional evaluation visit each time they presented clinical symptoms related to the defined CID classified by category… These evaluation visits were conducted 3 days (±1 day) after the initiation of the symptoms. At each additional visit, a clinical examination was performed, the type of CID was diagnosed and the start date (first day of symptoms), end date (last day of symptoms) and global severity (defined as mild, moderate or severe) of the disease were reported. A CID for which data were captured at the additional visits was termed a 'CID regular reported' and was thus diagnosed by a doctor during the CID. Occasionally, data were captured at the planned visits and the infection defined as a 'CID delayed reported', which refers to a CID diagnosed by a doctor on the basis of symptoms reported by the volunteers after the end of CID event… At each of the additional [visits], biological samples were taken for the identification of specific pathogens according to the type of CID. In addition, blood samples were also collected in a subset of volunteers to assess the same biological parameters as for planned visits… In addition, analyses of the microorganisms responsible for CIDs were performed by measuring the occurrence and amount of specific pathogens in biological samples… For URTI viruses, identification and quantification in nasal fluid samples were performed by quantitative reverse transcription-PCR. For bacteria identification and quantification, in case of LRTI in expectorations or throat swabs (the latter in case of sore throat) or in case of GITI in stools samples, a microscopical examination and Gram staining were performed and samples were cultured on enriched and specific media."

b) If not diagnosed by a physician/health professional, type of symptoms listed (e.g., sneezing, runny nose): NA

c) If not diagnosed by a physician/health professional, number and duration of symptoms used to define a CID episode (e.g., must have at least 2 symptoms within 2 consecutive days): NA

2) Duration of CIDs: Yes

a) How duration was determined (e.g., first to the last day of symptoms): Yes; "A new CID occurring after a previous CID of the same type was considered as a separate event only if there was at least 2 days between the two events… duration of CID (both cumulative and average duration per episode) [was] calculated from the first to the last day of symptoms" as defined in Table 1 for each CID.

3) Severity of CIDs: Yes

a) How it was determined (e.g., scoring system: mild, moderate, severe): Yes; "severity of CID assessed with a three-point scale (mild–moderate–severe, based on the degree of interference with the subject's daily activity)."

[63] The following measures used to assess the outcomes were considered:

1) Incidence of CIDs: Yes

a) Diagnosed by a physician/health professional: Yes; "when a participant was feeling sick, he/she reported to the main investigator who visited the patient within 12 hours… Diagnoses, clinical symptoms and duration were recorded by a physician according to the case report form."

b) If not diagnosed by a physician/health professional, type of symptoms listed (e.g., sneezing, runny nose): NA

c) If not diagnosed by a physician/health professional, number and duration of symptoms used to define a CID episode (e.g., must have at least 2 symptoms within 2 consecutive days): NA

2) Duration of CIDs: NR

a) How duration was determined (e.g., first to the last day of symptoms): NR; "incidence and severity (duration, intensity and maximal temperature) of winter pathologies…"

3) Severity of CIDs: NR

a) How it was determined (e.g., scoring system: mild, moderate, severe): NR; "incidence and severity (duration, intensity and maximal temperature) of winter pathologies…"

[64] "To calculate the adjusted sample size, we used an estimate of 0.1 as the intra-class correlation between household, the design effect was 1.05, and the adjusted sample size required was 638 accounting for 20% effect size and a 20% dropout. The number of households required to provide this sample was around 426 with the assumption that average household size is 1.5. This sample size was based on setting statistical significance at 0.05 and 80% power."

[65] "For the sample size estimation before the study, an average number of 3 CID events over a 3-month period were assumed in the control group, and a 15% relative decrease was expected in the active group (i.e., the expected average number of events was 2.55 in the active group). Using a Poisson regression with a 2-sided test at the 5% α-level assuming moderate overdispersion (scale parameter of 1.1), around 230 evaluable children were estimated to be needed in each arm with ≥80% power. Furthermore, a 15% dropout rate was taken into account. As products were randomized to families (each eligible child of a family was given the same product), the sample size had to be adjusted to account for the family cluster, that is, the possible dependence of the occurrence of an infectious episode within the eligible children of the same family. Assuming an intraclass correlation coefficient of 0.1, and an average number of eligible children per family of 1.2, a total of 276 children per arm were needed. In the same way, the sample size had to be adjusted to account for the dependence of the occurrence of an infectious episode among eligible children in the same day-care centers or preschools. Assuming an intraclass correlation coefficient of 0.01, and an average number of eligible children per day-care of 10, around 300 children were needed in each arm. Therefore, a total of 600 children were planned to be included in the study."

[66] "With an expected rate of 1.5 events over the 3 months in the control group, and using a Poisson regression with a moderate overdispersion, a 2-sided test (α=0.05), 80% power, and a 15% reduction in rate with a dropout rate of 5%, 500 volunteers had to be enrolled in each arm."

[67] "For the pilot study, no calculation of sample size could be performed and thus sample size was determined through a review of the literature."

[68] "For the confirmatory study, a sample size was calculated from the results of the pilot study: 145 volunteers per group were required to show a difference of 0.7 in converted antibody titres against H3N2 at a power of 80% (α= 0.05). Given the size of the randomisation blocks and premature withdrawals, it was planned to include in the study a total of 348 individuals." A total of 241 subjects were randomised in the confirmatory study.

[69] "With an expected rate of 1·5 events over 3 months in the control group and using a Poisson regression with a two-sided test at the 5% a-level and assuming moderate overdispersion, about 450 evaluable volunteers in each arm were expected to be needed to detect a 15% reduction rate with at least 80% power. A 5% dropout rate of volunteers was assumed and as such inclusion of approximately 500 volunteers in each arm was necessary."

[70] "Reporting bias by selective outcome reporting was prevented by the availability of the study protocol and prespecification of (primary and secondary) outcomes, and by adhering to these specifications."

[71] "The sensitivity analysis done by looking at regularly versus delayed reported CIDs" was NR as a planned analysis in the methods. In addition, "because the Poisson distribution of the primary parameter did not fully fit to the observed data, especially because of an excess of zeros (based on unexpected low CID frequency), a categorical ordinal model was performed on CID cumulated number in 3 classes (post hoc analysis): 0/subjects with no CID, 1 to 2/subjects with a medium number of CID (i.e., 1 or 2 episodes), and >2/subjects with more than 2 CIDs. In this model, odds assumption has been checked and could be used appropriately to the observed data."

[72] "Subjects were randomized to the treatment group… or control group (no study product) and stratified for sex and vaccinal status. Each group was randomly divided into three sub-groups (n=60). The study consisted of three consecutive 3-week periods to reduce the risk of an influenza epidemic hampering the results. The first group, comprising treatment and control subjects, was studied from week 1 to week 3; a second group was studied from week 4 to week 6 and a third group was studied from week 7 to week 9… There were no statistically significant differences between the sub-groups of the control group. Therefore, the three sub-groups of both the treatment group and the control group were analysed as one for each group." The combining of the data from the 3 sub-groups was NR as a planned analysis in the methods. In addition, it was NR whether there were any significant differences between the sub-groups of the treatment groups.

[73] "Missing data were replaced by the Last Value Carried Forward method using the last post-baseline value for one subject at the earlier time when appropriate… All analyses of primary and secondary analyses of outcomes were conducted on an [ITT] basis," which included all randomized subjects.

[74] "All volunteers who were randomized and received the study products were included in the ITT population and were used for all statistical analyses. The PP population was built-up with all volunteers from the ITT population without any major protocol
deviations during the intervention phase (product consumption period). As the number of subjects with ≥1 major protocol
deviation was low, the ITT and PP populations were close. Therefore, analysis on the PP was conducted on the primary main
outcome only… To avoid attrition bias according to the recommendations of the Cochrane Collaboration, the distribution of the missing data across the intervention groups and the magnitude compared with the effect size were assessed. Missing data were replaced by the last observation carried forward method using the last post baseline value for 1 subject at the previous time."

[75] "Analyses were performed on the ITT population."

[76] A per protocol analysis on 502 subjects who completed the study was conducted.

[77] "All analyses have been performed on the ITT population."

[78] "All volunteers who were randomised and received the study product were included in the ITT population, considered as the main population for statistical analysis."

[79] "Analyses were performed on the ITT population, which comprised all volunteers who were included, randomised to the groups and having received the study product. Analyses were also performed on the per protocol population."

[80] "Analysis was done as ITT."

[81] The NIH stated that "the questions on the assessment tool were designed to help reviewers focus on the key concepts for evaluating a study's internal validity. They are not intended to create a list that is simply tallied up to arrive at a summary judgment of quality… High risk of bias translates to a rating of poor quality. Low risk of bias translates to a rating of good quality… If a study has a "fatal flaw," then risk of bias is significant, and the study is of poor quality. Examples of fatal flaws in RCTs include high dropout rates, high differential dropout rates, no ITT analysis or other unsuitable statistical analysis (e.g., completers-only analysis)."

[82] The following were considered fatal flaws: significant difference between groups in the number of study products consumed; and the self-reporting of CIDs by parents.

[83] The following were considered fatal flaws: CID-related outcomes were not assessed as primary nor secondary outcomes, and instead were reported as part of adverse events; and the differential drop-out rate could not be calculated based on the information reported in the study.
